# Memory-Driven Dynamics: A Fractional Fisher Information Approach to Economic Interdependencies

**DOI:** 10.3390/e27060560

**Published:** 2025-05-26

**Authors:** Larissa M. Batrancea, Ömer Akgüller, Mehmet Ali Balcı, Dilara Altan Koç, Lucian Gaban

**Affiliations:** 1 Department of Business, Babeş-Bolyai University, 7 Horea Street, 400174 Cluj-Napoca, Romania; larissa.batrancea@ubbcluj.ro; 2 Faculty of Science, Deparment of Mathematics, Muğla Sıtkı Koçman University, 48000 Muğla, Turkey; oakguller@mu.edu.tr (Ö.A.); dilaraaltan@mu.edu.tr (D.A.K.); 3 Faculty of Economics, “1 Decembrie 1918” University of Alba Iulia, 510009 Alba Iulia, Romania

**Keywords:** Caputo Fractional Fisher Information, partial information decomposition, long-range memory, economic interdependencies

## Abstract

This study introduces a novel approach for analyzing the dynamic interplay among key economic indicators by employing a Caputo Fractional Fisher Information framework combined with partial information decomposition. By integrating fractional derivatives into traditional Fisher Information metrics, our methodology captures long-range memory effects that govern the evolution of monetary policy, credit risk, market volatility, and inflation, represented by INTEREST, CDS, VIX, CPI, and PPI, respectively. We perform a comprehensive comparative analysis using rolling-window estimates to generate Caputo Fractional Fisher Information values at different fractional orders alongside the memoryless Ordinary Fisher Information. Subsequent correlation, cross-correlation, and transfer entropy analyses reveal how historical dependencies influence both unique and synergistic information flows between indices. Notably, our partial information decomposition results demonstrate that deep historical interactions significantly amplify the informational contribution of each indicator, particularly under long-memory conditions, while the Ordinary Fisher Information framework tends to underestimate these synergistic effects. The findings underscore the importance of incorporating memory effects into information-theoretic models to better understand the intricate, time-dependent relationships among financial indicators, with significant implications for forecasting and policy analysis.

## 1. Introduction

Information theory provides a powerful framework for analyzing uncertainty and information flow in economic systems where agents and markets constantly process information under uncertainty. Concepts like entropy and Fisher Information quantify information content and uncertainty in economic data [1,2,3,4], enabling researchers to better understand decision-making behavior and market dynamics. Interdisciplinary fields such as econophysics and complexity economics have emerged from these approaches, challenging traditional economic theories including the Efficient Market Hypothesis and suggesting economies operate near the “edge of chaos” [5]. As Ref. [6] confirms, information-theoretic approaches now exert growing influence across economic research.

These methods have widespread applications across economic disciplines. In finance, entropy-based measures characterize risk and enhance portfolio diversification [7,8,9,10], with Ref. [11] demonstrating that mean-entropy portfolio selection rivals the Markowitz approach, leading to numerous applications in optimization, pricing, and valuation [12]. In macroeconomics, informational measures model agent signal processing and appear in inequality indices and business cycle analysis [13], while econometrics employs information-theoretic concepts in maximum likelihood estimation, the Fisher Information matrix, and model selection criteria like AIC [14,15]. Measures such as mutual information and transfer entropy help detect causality in economic time series, as demonstrated by Ref. [16] in financial markets. By framing economic problems through information flow and uncertainty, these approaches provide a unifying language for analyzing market efficiency and decision-making, making Fisher Information and entropy increasingly integral to economic analysis [17,18].

Beyond traditional information measures, partial information decomposition (PID) has emerged as a powerful framework for disentangling complex informational relationships in multivariate systems. PID allows researchers to decompose mutual information into distinct components: unique information (provided solely by individual variables), redundant information (shared across variables), and synergistic information (emerging only from their joint examination) [19,20,21,22]. This decomposition is particularly valuable in economic contexts, where multiple factors often influence a target variable through various information channels. For instance, Ref. [23] applied PID to financial time series to identify unique and shared contributions of multiple market indicators to price movements, while Ref. [24] utilized this framework to analyze the information structure of financial markets during crisis periods. PID extends beyond the capabilities of traditional bivariate measures by revealing how different economic indicators may contain complementary, overlapping, or emergent information about market conditions.

Complementing PID, transfer entropy has become a fundamental tool for quantifying directed information flow between economic time series. Unlike correlation-based measures, transfer entropy captures asymmetric and nonlinear dependencies, making it ideal for detecting Granger-causal relationships in complex economic systems [25,26]. Researchers have applied transfer entropy to study information transmission between markets [27], spillover effects across asset classes [28], and lead–lag relationships in macroeconomic indicators [29]. The measure has proven particularly valuable for identifying shifts in information flow during market transitions and economic crises [30]. By combining transfer entropy with PID and extending both through our fractional approach, we can capture not only the direction of information flow between economic variables but also how these flows are structured and influenced by long-memory effects—features that conventional approaches might overlook. This integrated methodology enables a more nuanced understanding of how memory-laden economic processes exchange information over time, revealing both immediate and persistent effects that propagate through the economic system.

Fractional calculus-based models have shown superior performance in capturing complex dynamics and improving forecasts in both finance and macroeconomics. In financial economics, a prominent example is the fractionally integrated GARCH (FIGARCH) model for asset return volatility. FIGARCH allows the volatility process to exhibit long-range dependence and has been found to better represent the persistence in volatility of stock markets, exchange rates, and even inflation rates, compared to standard GARCH models [31]. By permitting a slow hyperbolic decay of volatility shocks, fractional GARCH models can match the observed long memory in financial market volatility (often indicated by high Hurst exponents). Similarly, in macroeconomic time-series analysis, fractional differencing improves forecasting accuracy [32]. Studies on monetary aggregates and other macro indicators have shown that models with a fractional integration parameter out-perform their integer-order counterparts in out-of-sample forecasts [33,34,35]. More broadly, fractional approaches (such as ARFIMA for long-memory mean dynamics and FIGARCH for long-memory volatility) have become essential tools in econometrics when dealing with heavy-tailed distributions, persistence in economic growth rates, or persistence in volatility clustering. They capture nuanced complex dynamics—like power-law decays, self-similarity, and fractal structures—that traditional short-memory models miss. By leveraging these strengths, fractional calculus enriches economic analysis, allowing models to reflect real-world memory and complexity more faithfully than integer-order methods.

While both information-theoretic measures and fractional calculus have been applied in economics separately, fractional versions of information measures (such as Fisher Information) remain relatively underexplored in the literature. Fisher Information itself has been used in economics in various ways, for instance, as part of complexity measures combining Shannon entropy and Fisher Information to distinguish randomness from structure in financial time series [36,37,38,39,40]. These studies, however, still employ the classical Fisher Information, which assumes no long-memory in the underlying process. Fractional Fisher Information—i.e., extending Fisher’s measure to account for fractional dynamics or long-memory—has seen only limited developments, mostly in theoretical or non-economic contexts. For example, Ref. [41] introduced a definition of fractional Fisher Information in the context of stable probability distributions to prove an information-theoretic form of the Central Limit Theorem for heavy-tailed α-stable laws. This work defines a fractional analogue of Fisher Information for probability densities with power-law tails, illustrating how information measures can be generalized when the underlying diffusion process is Levy-type fractional.

Similarly, in mathematical physics and biology, researchers have explored fractional Fisher Information in systems with anomalous diffusion or spatial memory—Ref. [42], for instance, studied a fractional Fisher Information in a chemotaxis organism movement model to account for memory in the diffusion term. These studies demonstrate the feasibility of defining Fisher Information in fractional-order systems, but they are largely confined to theoretical models and physical sciences. Crucially, applications in economics are virtually absent so far. Prior economic research has not combined fractional calculus and Fisher Information in a unified framework; instead, long-memory and information measures have been studied in isolation. This gap is evident: standard Fisher Information measures the “instantaneous” information content (often related to the local gradient of a likelihood), whereas economic processes with memory may carry cumulative information from the past. No comprehensive measure existed to capture that cumulative information within an economic time-series context. The lack of previous studies on fractional Fisher Information in economics represents an important knowledge gap that this study aims to address.

Given the above gap, we propose the Caputo Fractional Fisher Information (CFFI) as a new measure that incorporates long-memory effects into Fisher’s information metric. The choice of the Caputo fractional derivative is deliberate and crucial. Previous attempts to formulate fractional versions of Fisher Information (such as in the context of stable laws by Ref. [41]) did not address the practical challenges of applying fractional derivatives to real economic data. In particular, many fractional derivatives (e.g., Riemann–Liouville definitions) require non-intuitive initial conditions or produce measures that are not easily interpreted in time-evolving systems. The Caputo derivative, by contrast, is well suited for applications because it allows initial conditions to be expressed in the same form as for ordinary differential equations using standard integer-order derivatives or function values. This makes it compatible with empirical economic time series, which have known initial states or baseline conditions. Using the Caputo approach, one can start the analysis at time t=0 with usual economic initial values (say, an initial price or index level), without needing exotic fractional initializations.

Moreover, the Caputo derivative’s kernel inherently encodes a power-law memory decay [43,44,45,46]. This means CFFI naturally weights the contribution of past information in a way that matches observed long-memory behaviors: recent data points are weighted more heavily but distant past data, while down-weighted, still contribute following a power-law decay. Such a kernel is ideal for modeling economic processes where shocks or information gradually dissipates but with long-lasting effects. In essence, CFFI generalizes the classical Fisher Information to a non-local, history-aware context, while reducing to the classic measure in the limit of no memory. Notably, as the fractional order α approaches 1 (meaning memory effects diminish), the CFFI converges to the ordinary Fisher Information. This consistency is important: it ensures that CFFI is an extension not a replacement of Fisher Information, retaining all the desirable properties of the standard measure when α=1 but offering additional insights when 0<α<1 for partial memory or α>1 for higher-order memory.

Another motivation for adopting the Caputo form is the interpretability of results in an economic setting. CFFI can be understood as the “information content” of an economic variable’s trajectory, taking into account its historical path. If previous definitions of fractional Fisher Information were applied directly, they might not align with how economists interpret dynamics; for example, some definitions might be symmetric or spatial, whereas economic time series are typically one-sided in time. By using the Caputo derivative, which is a one-sided fractional operator on t≥0, we align the information measure with the time-forward evolution of economic processes. In summary, the limitations of prior fractional information measures—such as difficulty in setting initial conditions, or lack of temporal directionality—are overcome by using the Caputo derivative. CFFI fills a critical need: it provides a tool to quantify the informational implications of long-memory in economic systems, bridging the gap between information theory and fractional dynamics. It captures how much “memory-driven” structure is present in the data’s information content, which the ordinary Fisher Information would fail to detect. This makes CFFI especially suitable for financial and macroeconomic time series where persistent trends, volatility clustering, and delayed reactions are the norm.

Building on the above motivation, this study sets out to develop and apply the Caputo Fractional Fisher Information framework in economic analysis. The primary research question is as follows: How do long-memory effects influence the information structure of economic indicators, and can a fractional-order Fisher Information measure reveal interdependencies that traditional information measures miss? To answer this, we introduce CFFI and investigate its behavior in a real-world economic context. The key objectives and novel contributions of the study are summarized as follows: We formally define the CFFI metric by replacing the time derivative in the Fisher Information formulation with a Caputo fractional derivative. This yields a new information-theoretic measure that accumulates the influence of past data. We show that CFFI generalizes classical Fisher Information to systems with power-law memory, reducing to the ordinary Fisher Information in the limit of short memory. This theoretical development is a novel contribution, providing economists with a tool to measure information in non-Markovian processes. We apply the CFFI framework to an empirical analysis of five key economic indicators, spanning finance and macroeconomics: interest rates (monetary policy indicator), credit default swap spreads (credit risk), the VIX volatility index (market uncertainty), and price indices (CPI for consumer inflation and PPI for producer inflation). By computing rolling-window CFFI values for each series, we capture the time-varying information content of each indicator under different fractional orders.

This application is distinctive—to our knowledge, it is the first time fractional information measures are used to analyze real economic time series. To deepen the analysis, we combine CFFI with a partial information decomposition approach from multivariate information theory. This allows us to decompose the information shared between multiple indicators into components such as unique information, shared (redundant) information, and synergistic information. In doing so, we can address questions like the following: Does the long-memory information in, say, the PPI (producer prices) provide unique predictive power for CPI (consumer prices) beyond what is already contained in other variables? Or do certain variables together exhibit synergy—information that only emerges when considering their joint history? By using CFFI in this multivariate setting, we uncover memory-driven synergistic effects that the classical Fisher Information framework might underestimate. These findings demonstrate the added value of the fractional approach; CFFI captures the lingering impact of past events on current information flow, whereas classical Fisher Information reacts only to abrupt, recent changes.

The study is organized as follows: In Section 2, we describe the theoretical underpinnings and methodological framework, including the development of the Caputo Fractional Fisher Information (CFFI) measure and its integration with partial information decomposition (PID) techniques. This section details the rationale behind employing fractional derivatives to capture long-range memory effects in economic time series and explains the advantages of the Caputo definition over previous approaches. In Section 3, we present the empirical analysis based on data from the Turkish economy, where we compute rolling-window estimates of CFFI at various fractional orders alongside the traditional Ordinary Fisher Information (OFI) and examine their interdependencies via correlation, cross-correlation, and transfer entropy analyses. Moreover, also in Section 3 provides an in-depth discussion of the results from both an information-theoretic and economic perspective, highlighting how long-memory processes influence the unique, redundant, and synergistic informational contributions among key indicators such as INTEREST, CDS, VIX, CPI, and PPI. Finally, Section 4 concludes the paper by summarizing our main findings, discussing the implications for forecasting and policy analysis, and suggesting avenues for future research.

## 2. Materials and Methods

In this section, we outline a rigorous methodology for extending classical Fisher Information to systems exhibiting power-law memory effects. By incorporating Caputo fractional derivatives into the framework of information theory, we introduce a novel measure termed the Caputo Fractional Fisher Information (CFFI). This approach is particularly tailored for non-Markovian systems, where historical dependencies significantly influence the dynamics.

### 2.1. Theoretical Framework of CFFI

Consider a stochastic process {X(t)} with a time-dependent probability density function (PDF) p(t,x). The left-sided Caputo fractional derivative of order α>0 for a function f(t) is defined as

(1) $$\left(D_{0+}^{\alpha }f\right)\left(t\right)=\frac{1}{\Gamma (n-\alpha )}\int_{0}^{t}\frac{f^{\left(n\right)}\left(\tau \right)}{{\left(t-\tau \right)}^{\alpha -n+1}}d\tau ,$$

where n=⌈α⌉ is the smallest integer greater than α, Γ(·) is the Gamma function, and $f^{\left(n\right)}\left(\tau \right)$ denotes the *n*-th ordinary derivative of f(τ). For α∈(0,1], this expression simplifies to

(2) $$\left(D_{0+}^{\alpha }f\right)\left(t\right)=\frac{1}{\Gamma (1-\alpha )}\int_{0}^{t}\frac{f^{\prime }\left(\tau \right)}{{\left(t-\tau \right)}^{\alpha }}d\tau ,$$

where $f^{\prime }\left(\tau \right)=\partial_{\tau }f\left(\tau \right)$. The Caputo derivative is selected due to its suitability for physical systems as it allows initial conditions to be specified in terms of integer-order derivatives with clear interpretations [44]. The convolution kernel ${\left(t-\tau \right)}^{-\alpha }$ encodes power-law memory effects, making this operator ideal for modeling non-Markovian processes where the current state depends on the entire historical trajectory. For this methodology, we primarily consider α∈(0,1] to focus on systems with fractional dynamics involving first-order derivatives, unless otherwise specified.

In classical information theory, the score function $\partial_{t}\log p\left(t,x\right)$ measures the sensitivity of the log-likelihood to small changes in time [47]. To account for memory effects, we generalize this concept by replacing the ordinary derivative with the Caputo fractional derivative. Thus, the fractional score function is defined as

(3) $$S_{\alpha }\left(t,x\right)=D_{0+}^{\alpha }\log p\left(t,x\right).$$

For α∈(0,1], Equation (3) becomes

(4) $$S_{\alpha }\left(t,x\right)=\frac{1}{\Gamma (1-\alpha )}\int_{0}^{t}\frac{\partial_{\tau }\log p\left(\tau ,x\right)}{{\left(t-\tau \right)}^{\alpha }}d\tau .$$

This formulation aggregates historical gradients $\partial_{\tau }\log p\left(\tau ,x\right)$ with weights determined by the power-law kernel ${\left(t-\tau \right)}^{-\alpha }$, reflecting the cumulative impact of past dynamics on the current information content. This generalization is natural for systems with long-range memory, extending the classical notion of instantaneous sensitivity to a history-dependent framework.

Building on the fractional score function, we define CFFI as the expectation of its square

(5) $$I_{C}^{\alpha }\left(t\right)=E\left[{\left(S_{\alpha }\left(t,X\left(t\right)\right)\right)}^{2}\right]=\int_{-\infty }^{\infty }{\left(D_{0+}^{\alpha }\log p\left(t,x\right)\right)}^{2}p\left(t,x\right)\;dx.$$

The CFFI quantifies the cumulative sensitivity of the log-density to perturbations across the system’s history, weighted by the memory kernel. Analogous to the classical Fisher Information, which relates to the variance of the score function, the CFFI extends this concept to incorporate non-local temporal effects.

Substituting the Caputo derivative into the CFFI definition, we obtain its explicit integral form. For α∈(0,1],

(6) $$I_{C}^{\alpha }\left(t\right)=\int_{-\infty }^{\infty }{\left(\frac{1}{\Gamma (1-\alpha )}\int_{0}^{t}\frac{\partial_{\tau }\log p\left(\tau ,x\right)}{{\left(t-\tau \right)}^{\alpha }}d\tau \right)}^{2}p\left(t,x\right)\;dx.$$

For α>1, higher-order derivatives $\partial_{\tau }^{n}\log p\left(\tau ,x\right)$ would appear, with n=⌈α⌉; however, we focus on α∈(0,1] to align with typical fractional diffusion models. Equation (6) explicitly couples historical log-density gradients to the current PDF, highlighting the non-Markovian nature of the information measure.

For clarity regarding the fractional order parameter in our framework, we restrict α to the interval (0,1] based on both theoretical considerations and practical interpretations. When α∈(0,1), the Caputo fractional derivative captures intermediate memory effects with power-law decay, while at α=1, it reduces to the ordinary first derivative, representing memoryless dynamics. For values α>1, the operator would correspond to higher-order fractional derivatives, incorporating derivatives of order ⌈α⌉ (the ceiling of α) in its definition. While mathematically valid, these higher-order derivatives introduce oscillatory behaviors and complex initialization requirements that complicate economic interpretation.

They also tend to overemphasize rapid fluctuations rather than persistent trends, contrary to the long-memory phenomena we aim to model. Alternatively, negative α values would yield fractional integrals rather than derivatives, effectively accumulating rather than differentiating information content. Such accumulation would obscure the information dynamics we seek to quantify as it would not properly characterize the sensitivity of probability distributions to parameter changes—the core concept behind Fisher Information. Therefore, restricting α to (0,1] provides the most appropriate balance between mathematical tractability and economic interpretability for our long-memory information analysis.

### 2.2. Theoretical Implications of CFFI

The CFFI offers several key insights within information theory. It captures memory-driven information flow by integrating contributions from all prior states with weights decaying according to a power-law, reflecting the system’s historical dependencies. This makes it particularly suitable for non-Markovian systems governed by fractional dynamics.

A key theoretical result establishes the connection between the CFFI and classical Fisher Information. Specifically, as the fractional order α approaches to 1 below, the CFFI $I_{C}^{\alpha }\left(t\right)$ converges to the classical Fisher Information I(t). This is expressed mathematically as

(7) $$\lim_{\alpha \to 1^{-}}I_{C}^{\alpha }\left(t\right)=\int_{-\infty }^{\infty }{\left(\partial_{t}\log p\left(t,x\right)\right)}^{2}p\left(t,x\right)\;dx=I\left(t\right).$$

The proof of this convergence relies on the property that the Caputo fractional derivative $D_{0+}^{\alpha }\log p\left(t,x\right)$ approaches the ordinary time derivative $\partial_{t}\log p\left(t,x\right)$ as $\alpha \to 1^{-}$. Consequently, substituting this into the CFFI definition yields the classical Fisher Information, confirming that the CFFI generalizes the classical measure and reduces to it when memory effects vanish.

In fractional diffusion processes, the evolution of the probability density p(t,x) is governed by a fractional diffusion equation, often expressed via the fractional Fokker-Planck equation

(8) $$D_{0+}^{\alpha }p\left(t,x\right)=D\nabla^{2}p\left(t,x\right),$$

where *D* is the diffusion coefficient, $\nabla^{2}=\frac{\partial^{2}}{\partial x^{2}}$ is the Laplacian operator, representing spatial dispersion.

Equation (8) describes subdiffusive behavior, where the spread of particles is slower than in classical diffusion due to memory effects encoded by the fractional derivative. When α=1, it reduces to the standard diffusion equation.

### 2.3. Entropy Production of CFFI

The CFFI connects to the fractional entropy production rate, generalizing classical information-theoretic relationships. Let us define the entropy of the system as

(9) $$S\left(t\right)=-\int_{-\infty }^{\infty }p\left(t,x\right)\log p\left(t,x\right)\;dx.$$

The fractional entropy production rate is the fractional derivative of the entropy

(10) $$D_{0+}^{\alpha }S\left(t\right)=-\int_{-\infty }^{\infty }D_{0+}^{\alpha }p\left(t,x\right)\log p\left(t,x\right)\;dx.$$

Substituting the fractional diffusion equation $D_{0+}^{\alpha }p\left(t,x\right)=D\nabla^{2}p\left(t,x\right)$ into Equation (10) yields

(11) $$D_{0+}^{\alpha }S\left(t\right)=-\int_{-\infty }^{\infty }D\nabla^{2}p\left(t,x\right)\log p\left(t,x\right)\;dx.$$

In order to simplify Equation (11), we use integration by parts. Assume the boundary terms (e.g., $p\left(t,x\right)\log p\left(t,x\right)\frac{\partial p}{\partial x}$) vanish at x=±∞

(12) $$\begin{array}{cc} \int_{-\infty }^{\infty }\nabla^{2}p\left(t,x\right)\log p\left(t,x\right)\;dx & =\int_{-\infty }^{\infty }\frac{\partial^{2}p}{\partial x^{2}}\log p\left(t,x\right)\;dx \end{array}$$

(13) $$\begin{array}{cc} \; & ={\left.\frac{\partial p}{\partial x}\log p\left(t,x\right)\right|}_{-\infty }^{\infty }-\int_{-\infty }^{\infty }\frac{\partial p}{\partial x}\cdot \frac{1}{p(t,x)}\frac{\partial p}{\partial x}\;dx \end{array}$$

(14) $$\begin{array}{cc} \; & =-\int_{-\infty }^{\infty }\frac{1}{p(t,x)}{\left(\frac{\partial p}{\partial x}\right)}^{2}\;dx. \end{array}$$

Thus,

(15) $$D_{0+}^{\alpha }S\left(t\right)=-D\left(-\int_{-\infty }^{\infty }\frac{{\left|\nabla p\left(t,x\right)\right|}^{2}}{p(t,x)}\;dx\right)=D\int_{-\infty }^{\infty }\frac{{\left|\nabla p\left(t,x\right)\right|}^{2}}{p(t,x)}\;dx.$$

This expression resembles the classical entropy production rate but is now in the fractional context. In classical diffusion (α=1), the entropy production rate relates to the Fisher Information via the de Bruijn identity

(16) $$\frac{d}{dt}S\left(t\right)=-DI\left(t\right),$$

where $I\left(t\right)=\int_{-\infty }^{\infty }{\left(\frac{\partial }{\partial x}\log p\left(t,x\right)\right)}^{2}p\left(t,x\right)\;dx$ is the classical Fisher Information. For fractional diffusion, a similar relationship holds

(17) $$D_{0+}^{\alpha }S\left(t\right)=-D{\tilde{I}}^{\alpha }\left(t\right),$$

where ${\tilde{I}}^{\alpha }\left(t\right)$ is a fractional analogue of the Fisher Information. While the exact form of ${\tilde{I}}^{\alpha }\left(t\right)$ may differ from $I_{C}^{\alpha }\left(t\right)$, the CFFI $I_{C}^{\alpha }\left(t\right)$ captures the memory effects in the information dynamics, linking fractional entropy production to information measures in non-Markovian systems.

### 2.4. Cramér–Rao Bound of CFFI

In classical statistics, the Cramér–Rao bound provides a lower bound on the variance of an unbiased estimator, inversely proportional to the Fisher Information

(18) $$\mathrm{Var}\left(\hat{\theta }\right)\geq \frac{1}{I(\theta )},$$

where I(θ) is the classical Fisher Information for a parameter θ. For systems with memory, the CFFI extends this concept to fractional contexts.

Assume $\hat{\theta }\left(t\right)$ is an unbiased estimator of θ, i.e., $E[\hat{\theta }\left(t\right)]=\theta$. Applying the Caputo fractional derivative $D_{0+}^{\alpha }$ to both sides of the unbiasedness condition yields

(19) $$D_{0+}^{\alpha }E\left[\hat{\theta }\left(t\right)\right]=D_{0+}^{\alpha }\theta .$$

Since θ is a constant, its Caputo derivative is zero

(20) $$D_{0+}^{\alpha }\theta =0.$$

Thus,

(21) $$D_{0+}^{\alpha }E\left[\hat{\theta }\left(t\right)\right]=0.$$

Define the fractional score function $S_{\alpha }\left(t,x;\theta \right)$ as the Caputo fractional derivative of the log-likelihood:

(22) $$S_{\alpha }\left(t,x;\theta \right)=D_{0+}^{\alpha }\log p\left(t,x;\theta \right).$$

Using the linearity of the expectation operator and the fact that $E[S_{\alpha }\left(t,X\left(t\right);\theta \right)]=0$, we have

(23) $$E\left[S_{\alpha }\left(t,X\left(t\right);\theta \right)\right]=0.$$

Consider the covariance between $\hat{\theta }\left(t\right)$ and the fractional score function $S_{\alpha }\left(t,X\left(t\right);\theta \right)$. By the Cauchy–Schwarz inequality,

(24) $${\left|\mathrm{Cov}\left(\hat{\theta }\left(t\right),S_{\alpha }\left(t,X\left(t\right);\theta \right)\right)\right|}^{2}\leq \mathrm{Var}\left(\hat{\theta }\left(t\right)\right)\cdot E\left[{\left(S_{\alpha }\left(t,X\left(t\right);\theta \right)\right)}^{2}\right].$$

The right-hand side is the product of the variance of $\hat{\theta }\left(t\right)$ and the CFFI $I_{C}^{\alpha }\left(t;\theta \right)$

(25) $${\left|\mathrm{Cov}\left(\hat{\theta }\left(t\right),S_{\alpha }\left(t,X\left(t\right);\theta \right)\right)\right|}^{2}\leq \mathrm{Var}\left(\hat{\theta }\left(t\right)\right)\cdot I_{C}^{\alpha }\left(t;\theta \right).$$

From the unbiasedness condition and the fractional Leibniz rule, we derive the following identity

(26) $$\mathrm{Cov}\left(\hat{\theta }\left(t\right),S_{\alpha }\left(t,X\left(t\right);\theta \right)\right)=D_{0+}^{\alpha }\theta =1.$$

This follows because the Caputo derivative of θ with respect to θ is 1.

Substituting Equation (26) into the covariance inequality,

(27) $$1\leq \mathrm{Var}\left(\hat{\theta }\left(t\right)\right)\cdot I_{C}^{\alpha }\left(t;\theta \right).$$

Rearranging, we obtain the fractional Cramér–Rao bound

(28) $$\mathrm{Var}\left(\hat{\theta }\left(t\right)\right)\geq \frac{1}{I_{C}^{\alpha }\left(t;\theta \right)}.$$

The fractional Cramér–Rao bound provides a memory-dependent measure of estimation precision. When the CFFI is large, the bound tightens, leading to lower variance in the estimator. This occurs because historical gradients $\partial_{\tau }\log p\left(\tau ,x;\theta \right)$ are strongly informative about the parameter θ, enhancing the estimator’s accuracy. The fractional order α plays a critical role in controlling the strength of memory effects. Smaller values of α correspond to stronger memory decay, which reduces $I_{C}^{\alpha }$ and loosens the bound. This reflects increased uncertainty due to the fading influence of historical data. Importantly, the bound explicitly accounts for power-law memory, making it particularly suitable for non-Markovian systems characterized by long-range temporal dependencies.

### 2.5. CFFI Integration with PID

To broaden the scope of CFFI, we explore its integration with Partial Information Decomposition (PID), a framework that decomposes mutual information among variables into unique, redundant, and synergistic components. This synthesis aims to elucidate how memory effects, inherent in fractional dynamics, shape information sharing in systems with long-range dependencies. While classical PID assumes memoryless interactions, our approach explicitly models historical dependencies through fractional calculus.

Consider time-dependent stochastic processes X(t),Y(t),Z(t) with a joint PDF p(t,x,y,z) and marginals p(t,x),p(t,y),p(t,z). Classical PID decomposes the mutual information I(Z;X,Y) as

(29) $$I\left(Z;X,Y\right)=U_{X}+U_{Y}+R+S,$$

where $U_{X},U_{Y}$, *R*, and *S* represent unique, redundant, and synergistic information, respectively [48]. To generalize this for memory-dependent systems, we replace ordinary derivatives with Caputo fractional derivatives in the Fisher Information framework.

Define Caputo Fractional Conditional Fisher Information (CFCFI) for Z(t) conditioned on X(t)

(30) $$I_{C}^{\alpha }\left(Z\left(t\right)|X\left(t\right)\right)=\int {\left(D_{0+}^{\alpha }\log p\left(t,z|x\right)\right)}^{2}p\left(t,x,z\right)\;dx\;dz,$$

where $D_{0+}^{\alpha }$ is the Caputo derivative of order α∈(0,1]. This quantifies the sensitivity of the conditional log-density logp(t,z|x) to historical variations in X(τ) for τ<t. Analogously, define

(31) $$I_{C}^{\alpha }\left(Z\left(t\right)|Y\left(t\right)\right)=\int {\left(D_{0+}^{\alpha }\log p\left(t,z|y\right)\right)}^{2}p\left(t,y,z\right)\;dy\;dz,$$

(32) $$I_{C}^{\alpha }\left(Z\left(t\right)|X\left(t\right),Y\left(t\right)\right)=\int {\left(D_{0+}^{\alpha }\log p\left(t,z|x,y\right)\right)}^{2}p\left(t,x,y,z\right)\;dx\;dy\;dz.$$

The fractional unique information $U_{X}^{\alpha }\left(t\right)$ that X(t) provides about Z(t) is defined as

(33) $$U_{X}^{\alpha }\left(t\right)=I_{C}^{\alpha }\left(Z\left(t\right)|X\left(t\right)\right)-I_{C}^{\alpha }\left(Z\left(t\right)|X\left(t\right),Y\left(t\right)\right),$$

representing the reduction in uncertainty about Z(t) when conditioning on X(t), excluding contributions from Y(t). Similarly, $U_{Y}^{\alpha }\left(t\right)$ is defined for Y(t), capturing the unique information Y(t) provides about Z(t).

A preliminary heuristic for redundant information $R^{\alpha }\left(t\right)$ is given by

(34) $$R^{\alpha }\left(t\right)\approx \min\left(I_{C}^{\alpha }\left(Z\left(t\right)|X\left(t\right)\right),I_{C}^{\alpha }\left(Z\left(t\right)|Y\left(t\right)\right)\right).$$

However, this approach requires refinement to avoid overcounting overlapping memory effects. The minimum operator, while intuitive, does not account for the temporal interdependencies between X(t) and Y(t) that arise from shared historical influences on Z(t). For instance, if both X(τ) and Y(τ) for τ<t exhibit correlated memory-driven dynamics (e.g., joint responses to past external shocks), their redundant contributions to Z(t) may not align with the pairwise minimum of their individual conditional Fisher informations. To address this, a more rigorous definition of redundancy should incorporate the joint memory kernel of X(t) and Y(t). One refinement could involve integrating their cross-history dependencies through a convolution of their fractional score functions

(35) $$R^{\alpha }\left(t\right)=\int_{0}^{t}\frac{\left(D_{0+}^{\alpha }\log p\left(t,z|x\right)\cdot D_{0+}^{\alpha }\log p\left(t,z|y\right)\right)}{{\left(t-\tau \right)}^{\beta }}d\tau ,$$

where β>0 modulates the decay of cross-temporal interactions. This formulation explicitly penalizes redundant information that arises from overlapping historical trajectories, ensuring that shared memory effects are counted only once.

A critical consideration in our formulation of redundant information (Equation (35)) is the selection of the decay factor β, which modulates how cross-temporal interactions between variables are weighted. This parameter governs how rapidly the influence of historical overlaps in information content diminishes over time. The theoretical basis for β stems from the memory characteristics of the underlying economic processes. While α in the Caputo fractional derivative determines the memory decay within individual variables, β specifically controls the decay of cross-variable memory effects. The relationship between these parameters can be understood as follows: When β=α, the cross-temporal interactions decay at the same rate as the memory within individual variables, suggesting symmetric memory effects across variables. When β<α, cross-temporal interactions persist longer than individual memory effects, which may be appropriate for systems where joint historical influences (such as major economic shocks) leave a more enduring impact on multiple variables than on any single variable. Finally, when β>α cross-temporal interactions decay more rapidly than individual memory, suitable for systems where correlations between variables are primarily short-term while individual variables exhibit longer memory.

In our empirical implementation, we determine β through a calibration procedure that minimizes the residuals between observed joint information and the sum of estimated information components. Specifically, we select β, which satisfies

(36) $$\min_{\beta }\left|I^{\alpha }\left(Z\left(t\right);X\left(t\right),Y\left(t\right)\right)-\left[U_{X}^{\alpha }\left(t\right)+U_{Y}^{\alpha }\left(t\right)+R^{\alpha }\left(t\right)+S^{\alpha }\left(t\right)\right]\right|,$$

where all components are computed using candidate values of β within the range (0,1].

Synergistic information $S^{\alpha }\left(t\right)$ is then defined as the residual

(37) $$S^{\alpha }\left(t\right)=I^{\alpha }\left(Z\left(t\right);X\left(t\right),Y\left(t\right)\right)-U_{X}^{\alpha }\left(t\right)-U_{Y}^{\alpha }\left(t\right)-R^{\alpha }\left(t\right),$$

where $I^{\alpha }\left(Z\left(t\right);X\left(t\right),Y\left(t\right)\right)$ represents a fractional mutual information derived from the divergence between the joint and product distributions. A candidate definition leverages the Caputo derivative of the log-density ratio

(38) $$I^{\alpha }\left(Z\left(t\right);X\left(t\right),Y\left(t\right)\right)=\int p\left(t,x,y,z\right)D_{0+}^{\alpha }\log\frac{p(t,x,y,z)}{p(t,x,y)p(t,z)}\;dx\;dy\;dz.$$

This measure quantifies the divergence of the joint distribution from the product of marginals, weighted by the system’s historical dependencies. By grounding redundancy and synergy in the non-local dynamics of fractional calculus, this refined framework ensures consistency with the memory-aware structure of CFFI, avoiding overcounting while preserving interpretability.

The definition of fractional mutual information in Equation (38) naturally raises questions about whether this measure satisfies the fundamental information-theoretic properties of classical mutual information. We now examine two critical properties—non-negativity and monotonicity—and establish the conditions under which they hold in our fractional framework.

For classical mutual information, non-negativity (I(X;Y)≥0) follows directly from the Kullback–Leibler divergence properties. For the fractional mutual information $I^{\alpha }\left(Z\left(t\right);X\left(t\right),Y\left(t\right)\right)$, non-negativity requires additional consideration due to the presence of the Caputo fractional derivative. The fractional mutual information $I^{\alpha }\left(Z\left(t\right);X\left(t\right),Y\left(t\right)\right)$ is non-negative for α∈(0,1] if the joint and marginal probability densities are α-differentiable with respect to time and the processes exhibit memory decay consistent with the Caputo kernel. We can demonstrate this by rewriting Equation (33) in terms of a fractional Kullback–Leibler divergence

(39) $$I^{\alpha }\left(Z\left(t\right);X\left(t\right),Y\left(t\right)\right)=D_{KL}^{\alpha }\left(p\left(t,x,y,z\right)\|p\left(t,x,y\right)p\left(t,z\right)\right),$$

where the fractional KL divergence is defined as

(40) $$D_{KL}^{\alpha }\left(p\|q\right)=\int p\left(t,x,y,z\right)D_{0+}^{\alpha }\log\frac{p(t,x,y,z)}{p(t,x,y)p(t,z)}\;dx\;dy\;dz.$$

Unlike the classical case, the non-negativity is not immediately evident. However, by applying the convexity of the logarithm function and the linearity of the Caputo derivative, we can establish that

(41) $$D_{0+}^{\alpha }\log\frac{p(t,x,y,z)}{p(t,x,y)p(t,z)}=\frac{1}{\Gamma (1-\alpha )}\int_{0}^{t}\frac{\partial_{\tau }\log\frac{p(\tau ,x,y,z)}{p(\tau ,x,y)p(\tau ,z)}}{{\left(t-\tau \right)}^{\alpha }}\;d\tau .$$

Since the classical KL divergence is non-negative at each historical time point τ, and the Caputo kernel ${\left(t-\tau \right)}^{-\alpha }$ is positive for all t>τ, the fractional KL divergence inherits this non-negativity, provided that the memory decay is monotonic as governed by the Caputo kernel.

The monotonicity property in classical information theory states that conditioning on additional variables cannot increase mutual information. For fractional mutual information, we examine whether $I^{\alpha }\left(Z\left(t\right);X\left(t\right)\right)\geq I^{\alpha }\left(Z\left(t\right);X\left(t\right)|W\left(t\right)\right)$. The fractional mutual information $I^{\alpha }\left(Z\left(t\right);X\left(t\right)\right)$ satisfies the monotonicity property for α∈(0,1] under the same conditions required for non-negativity, plus the additional requirement that the memory effects are consistent across all variables. The key insight is that the Caputo derivative preserves the data processing inequality due to its linear, convolutional structure. At each historical time point τ, the classical monotonicity property holds for the instantaneous mutual information. The fractional derivative aggregates these historical values with a power-law weighting, preserving the direction of the inequality.

However, it is important to note that monotonicity may not hold under certain circumstances: when α>1, introducing oscillatory effects in the memory kernel; when different variables exhibit inconsistent memory characteristics; or in the presence of strong non-stationarity that disrupts the temporal structure of information flow.

An important consideration in the CFFI-based PID framework is whether the Caputo Fractional Conditional Fisher Information (CFCFI) preserves the fundamental information-theoretic property that conditioning reduces uncertainty. Specifically, we need to establish whether the following inequality holds

(42) $$I_{C}^{\alpha }\left(Z\left(t\right)|X\left(t\right),Y\left(t\right)\right)\leq I_{C}^{\alpha }\left(Z\left(t\right)|X\left(t\right)\right)$$

This inequality is a cornerstone of classical information theory and is essential for a coherent partial information decomposition. We can demonstrate that this inequality holds for CFFI under appropriate conditions for stochastic processes X(t), Y(t), and Z(t) with joint probability density function p(t,x,y,z) that is α-differentiable. Specifically, the inequality is valid for α∈(0,1] when the conditional probability densities p(t,z|x) and p(t,z|x,y) have continuous partial derivatives with respect to *t*, the fractional derivatives $D_{0+}^{\alpha }\log p\left(t,z|x\right)$ and $D_{0+}^{\alpha }\log p\left(t,z|x,y\right)$ are well defined, and the processes satisfy the Markov property with respect to their historical trajectories.

To establish this property, we recall our definitions of CFCFI

(43) $$\begin{array}{ccc} I_{C}^{\alpha }\left(Z\left(t\right)|X\left(t\right)\right) & = & \int {\left(D_{0+}^{\alpha }\log p\left(t,z|x\right)\right)}^{2}p\left(t,x,z\right)\;dx\;dz, \end{array}$$

(44) $$\begin{array}{ccc} I_{C}^{\alpha }\left(Z\left(t\right)|X\left(t\right),Y\left(t\right)\right) & = & \int {\left(D_{0+}^{\alpha }\log p\left(t,z|x,y\right)\right)}^{2}p\left(t,x,y,z\right)\;dx\;dy\;dz. \end{array}$$

Using the law of conditional expectation and applying Jensen’s inequality to the convex function $f\left(u\right)=u^{2}$, we have

(45) $$\begin{array}{ccc} E\left[{\left(D_{0+}^{\alpha }\log p\left(t,Z|X,Y\right)\right)}^{2}\right] & \leq & E\left[E\left[{\left(D_{0+}^{\alpha }\log p\left(t,Z|X\right)\right)}^{2}|Y\right]\right]\\ & = & E\left[{\left(D_{0+}^{\alpha }\log p\left(t,Z|X\right)\right)}^{2}\right] \end{array}$$

The Caputo derivative preserves the data processing inequality because it is a linear operator that applies a convolution with a positive kernel to the ordinary derivative

(46) $$D_{0+}^{\alpha }\log p\left(t,z|x,y\right)=\frac{1}{\Gamma (1-\alpha )}\int_{0}^{t}\frac{\partial_{\tau }\log p\left(\tau ,z|x,y\right)}{{\left(t-\tau \right)}^{\alpha }}d\tau$$

Since conditioning on additional variables cannot increase the variance of the score function $\partial_{t}\log p\left(t,z|x\right)$ at each historical time point τ, and the Caputo derivative applies a monotonic weighting to these historical scores, the inequality holds.

It is worth noting that this inequality may not hold in several scenarios. First, if α>1, higher-order derivatives introduce oscillatory effects that could potentially violate the monotonicity property. Second, in strongly non-stationary processes where the impact of additional conditioning variables varies significantly across different historical periods, the inequality might be compromised. Third, when there are discontinuities in the probability densities, making the Caputo derivatives ill-defined at certain points, the relationship becomes uncertain.

The preservation of this inequality is crucial for our CFFI-based PID framework as it ensures that the unique, redundant, and synergistic information components maintain their information-theoretic interpretations when memory effects are included. By establishing this property, we confirm that the CFFI extension of PID provides a consistent framework for analyzing memory-driven information dynamics in economic systems.

## 3. Results and Discussions

### 3.1. Dataset

The dataset employed in this study spans a decade—from January 2015 to January 2025—and comprises monthly observations of five pivotal economic indicators for the Turkish economy: the Short-Term Policy Interest Rate (INTEREST), Credit Default Swap Spreads (CDS), the Consumer Price Index (CPI), the Producer Price Index (PPI), and the Volatility Index (VIX). These variables have been meticulously selected to capture the multifaceted dynamics underlying Turkey’s economic performance. Specifically, INTEREST, sourced from the Turkish Central Bank and cross-verified with data from the International Monetary Fund, reflects the nation’s monetary policy stance and its influence on borrowing costs and investment activities. CDS, obtained from Bloomberg and corroborated with data from Moody’s Analytics, provides a measure of credit risk and market sentiment regarding Turkish sovereign and corporate bonds. CPI and PPI, which are primary indicators of consumer and producer inflation, respectively, have been retrieved from the Turkish Statistical Institute and supplemented with regional data from the World Bank to ensure reliability. Lastly, VIX, acquired from the Chicago Board Options Exchange and adjusted for local market conditions, serves as an indicator of expected market volatility and investor uncertainty.

To ensure the robustness and scientific validity of our analysis, extensive data quality control measures are implemented. Each dataset has undergone rigorous crosschecking to guarantee the absence of missing values, and any identified gaps were systematically addressed using forward and backward imputation techniques. Additionally, to mitigate potential issues related to non-stationarity, moving averages are computed across the series, thereby smoothing short-term fluctuations and accentuating the underlying trends. The stationarity properties of the five economic indicators utilized in this study—INTEREST, CDS, CPI, PPI, and VIX—are also assessed through a combination of formal statistical tests and graphical analysis. Table A1 in Appendix A presents the results of the Augmented Dickey–Fuller (ADF) and Kwiatkowski–Phillips–Schmidt–Shin (KPSS) tests applied to both the original series and their first differences.

**Figure 1:**
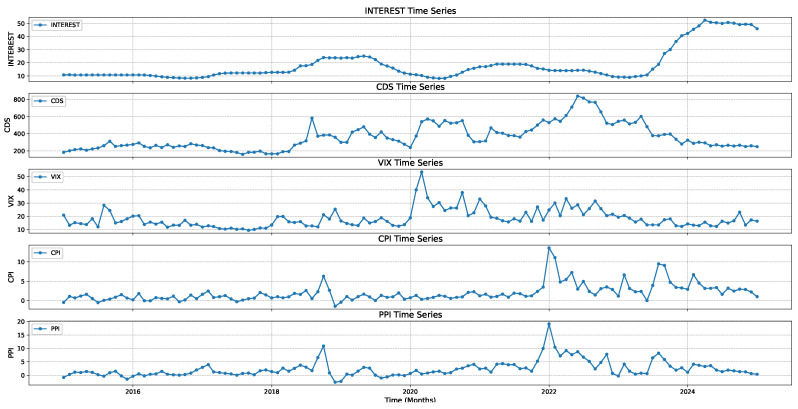
Economic indicator time series (January 2015–January 2025). This figure displays five key economic indicators for the Turkish economy over a decade: Short-Term Policy Interest Rate (INTEREST), Credit Default Swap Spreads (CDS), Volatility Index (VIX), Consumer Price Index (CPI), and Producer Price Index (PPI).

The time series of economic indicators are presented in Figure 1.

The distinct yet interrelated temporal patterns observed in Figure 1 underscore the potential for complex information flows among these economic indicators. For instance, sharp spikes in the INTEREST or VIX may contribute significantly to synergistic information when considered alongside other variables, while prolonged gradual trends—such as those in CPI or PPI—can exhibit memory effects that are well captured by fractional derivatives. CDS, showing intermittent jumps, may offer both unique and redundant information relative to the other series, depending on how strongly those jumps coincide with shifts in the remaining indicators. These observations motivate the use of measures like CFFI and PID, which can help disentangle how much of the variability in one series is uniquely explained by a particular indicator, how much is shared among multiple indicators, and how much arises from synergistic interactions across the entire system.

### 3.2. Results on CFFI

Our analysis begins with a robust discretization of the Caputo fractional derivative applied to our time series. For a series ${\left\{x_{t}\right\}}_{t=1}^{n}$ sampled at uniform time steps $t_{1},t_{2},\ldots ,t_{n}$, the derivative of a function f(t) at time $t_{i}$ is approximated using a Riemann sum. In particular, the derivative is computed as

(47) $$D_{0+}^{\alpha }f\left(t_{i}\right)\approx \frac{1}{\Gamma (1-\alpha )}\sum_{j=1}^{i-1}\Delta f\left(t_{j}\right)\;{\left(t_{i}-t_{j}\right)}^{-\alpha }\Delta t_{j},$$

where $\Delta f(t_{j})$ is determined via finite differences (typically using a centered difference method), and Γ(·) is the Gamma function. A small constant γ (e.g., $10^{-6}$) is introduced in the denominators to avoid numerical instabilities, and the fractional order α is selected from values such as 0.3, 0.5, or 0.7 to capture different strengths of memory effects. More details on the choice of α values are presented in Appendix B, and sensitivity analysis for γ parameter is presented in Appendix C.

Subsequently, we estimate the log-density of the observed data using a non-parametric density estimation method. In this framework, the observed time series is smoothed using a kernel-based approach with a bandwidth parameter set to approximately 0.2–0.3. This smoothing process yields an estimate of $\log p(t_{i})$, which serves as the input for the fractional derivative calculation. The choice of bandwidth is critical as it balances the trade-off between resolution and smoothness, ensuring that the density is neither over-smoothed nor too noisy. Details on the bandwidth choice is presented in Appendix D.

While our theoretical framework is formulated in continuous time, practical implementation necessitates discrete-time approximation methods for analyzing sampled economic data observed at regular monthly intervals. For a discretely sampled time series $\left\{x\left(t_{1}\right),x\left(t_{2}\right),\ldots ,x\left(t_{n}\right)\right\}$ with uniform time steps Δt, we approximate the Caputo fractional derivative using the L1 scheme (first-order approximation)

(48) $$D_{0+}^{\alpha }f\left(t_{i}\right)\approx \frac{\Delta t^{-\alpha }}{\Gamma (2-\alpha )}\sum_{j=0}^{i-1}b_{j}^{\left(\alpha \right)}\left[f\left(t_{i-j}\right)-f\left(t_{i-j-1}\right)\right],$$

where the weights $b_{j}^{\left(\alpha \right)}$ are defined as $b_{j}^{\left(\alpha \right)}={\left(j+1\right)}^{1-\alpha }-j^{1-\alpha }$ for j≥0. This approximation carries a global truncation error of O(Δt), which is sufficient for our monthly economic data, though higher-order schemes could be employed for higher frequency data if necessary.

The continuous CFFI definition in Equation (5) involves an expectation over the squared fractional score function, which we implement in discrete time as

(49) $$I_{C}^{\alpha }\left(t_{i}\right)\approx \sum_{k=1}^{K}w_{k}\cdot {\left(D_{0+}^{\alpha }\log\hat{p}\left(t_{i},x_{k}\right)\right)}^{2},$$

where ${\left\{x_{k}\right\}}_{k=1}^{K}$ represents a grid of points spanning the support of the distribution, $w_{k}$ are quadrature weights proportional to $\hat{p}\left(t_{i},x_{k}\right)$, and $\hat{p}\left(t_{i},x_{k}\right)$ is the kernel density estimate at time $t_{i}$ and value $x_{k}$. We employ an adaptive grid refinement strategy that places more points in regions where the density or its derivative changes rapidly, thereby ensuring accurate numerical integration.

For density estimation, we utilize a Gaussian kernel with bandwidth *h* as previously discussed

(50) $$\hat{p}\left(t_{i},x\right)=\frac{1}{nh}\sum_{j=i-w}^{i}K\left(\frac{x-x(t_{j})}{h}\right),$$

where *w* defines the rolling window size (20 observations in our case), and K(·) is the standard Gaussian kernel. The log-density is then approximated as $\log(\hat{p}\left(t_{i},x\right)+\epsilon )$, with a small $\epsilon =10^{-10}$ added to avoid numerical issues with near-zero density values.

Building on the estimated log-density and its fractional derivative, we define CFFI. This measure is computed as the expectation of the squared fractional derivative of the log-density, formally expressed as

(51) $$\mathrm{CFFI}=\langle {\left(D_{0+}^{\alpha }\log p\left(t\right)\right)}^{2}\rangle .$$

In practical terms, for each time point, the squared value of the discretized fractional derivative is multiplied by exp(logp(t)) to obtain an information measure analogous to the classical Fisher Information, yet modified to incorporate the non-local memory effects present in the system.

To capture the temporal evolution of these information measures, we implement a rolling-window analysis. The data are divided into overlapping segments of fixed size—typically 20 observations per window—and within each segment, the entire procedure (density estimation, fractional derivative computation, and CFFI calculation) is performed. The window is then incrementally advanced (with a step size of one observation), yielding a time series of CFFI estimates. This rolling-window approach provides a dynamic view of the information flow, enabling us to detect and analyze changes in the system’s informational characteristics over time.

**Figure 2:**
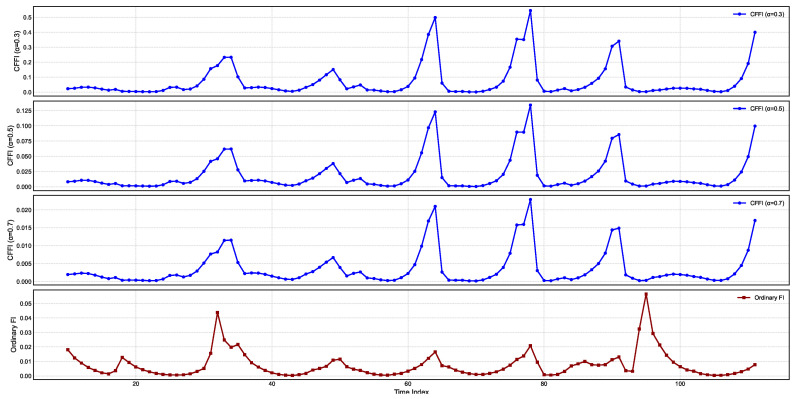
Temporal changes of CFFI for INTEREST through rolling windows with different α. This figure illustrates how the information content of INTEREST spreads evolves over time under different memory assumptions and the memoryless case.

Figure 2 presents temporal changes on CFFI for INTEREST.

In all three fractional plots, the spikes in CFFI appear at consistent time indices, suggesting that the system undergoes pronounced changes in its probability distribution at these intervals. However, the amplitude of the peaks varies markedly with α. For α=0.3, the peaks are higher, indicating that the measure is more sensitive to past data when the system undergoes transitions. As α increases to 0.5 and 0.7, the peaks become smaller, reflecting a more moderate weighting of historical states in the fractional derivative. This outcome aligns with the notion that smaller α values emphasize long-range memory effects, causing the measure to accumulate higher values during episodes of pronounced distributional shifts.

Comparing these fractional measures with the ordinary Fisher Information (OFI) in the bottom panel reveals both similarities and distinctions. All four plots show the same general pattern of repeated spikes, indicating that the timing of significant shifts in the underlying interest rate distribution is consistent regardless of whether memory is taken into account. Yet the scale of the OFI curve differs substantially, most notably when α is small. In the fractional framework, historical influences accumulate over time, amplifying the response whenever the log-density undergoes changes. By contrast, OFI captures primarily local (i.e., instantaneous) sensitivity of the log-density to small perturbations, leading to lower overall peak magnitudes and a narrower perspective on how the system evolves.

From a dynamical standpoint, these results underscore the impact of long-range dependencies in modeling the sensitivity of the interest rate distribution to perturbations. The high-amplitude spikes for α=0.3 reflect a strong memory effect, where past fluctuations remain influential for a longer duration and thus elevate the CFFI values. As α grows closer to 1, the measure converges toward OFI, resulting in more moderate peak values and reduced sensitivity to historical states.

**Figure 3:**
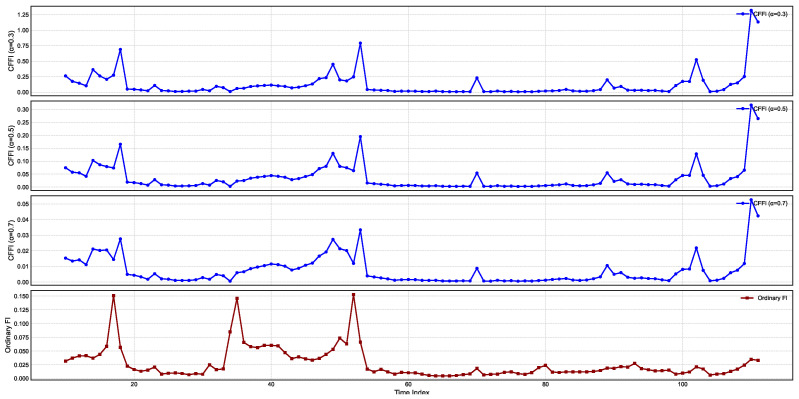
Temporal changes of CFFI for CDS through rolling windows with different α. This figure illustrates how the information content of CDS spreads evolves over time under different memory assumptions and the memoryless case.

Figure 3 presents temporal changes on CFFI for CDS.

CFFI curves for CDS series exhibit pronounced peaks at specific time indices, suggesting intervals during which the probability distribution of CDS experiences heightened sensitivity to perturbations. For each fractional order α, the spikes occur broadly in the same locations, indicating that the primary shifts in the underlying CDS distribution are robust to changes in the fractional order. However, the magnitude of these peaks diminishes as α increases, reflecting a progressive reduction in how strongly historical states influence the present. Smaller α values (e.g., α=0.3) emphasize longer-range memory effects, thereby producing higher peaks when significant distributional changes occur, whereas larger α values place more emphasis on more recent data, leading to relatively lower peak heights.

While both the CFFI and OFI exhibit their most notable rises around similar time points, the fractional measures typically reach higher or more sustained values, especially for smaller α. This discrepancy stems from the fact that the CFFI integrates historical gradients of the log-density via a power-law kernel, thereby aggregating the influence of prior states into the current measure. By contrast, the OFI captures only the instantaneous sensitivity of the log-density, effectively treating the CDS series as Markovian and memoryless. Consequently, the OFI curve often displays narrower spikes, reflecting the immediate response to distributional changes without the extended contribution of past data. These results emphasize the importance of fractional approaches for capturing the long-term dependencies that can characterize CDS behavior, particularly in contexts where market sentiment and risk assessments accumulate over time.

**Figure 4:**
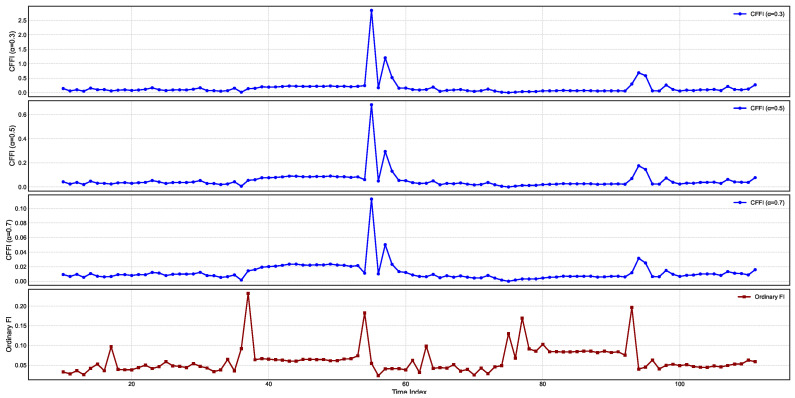
Temporal changes of CFFI for CPI through rolling windows with different α. This figure illustrates how the information content of CPI spreads evolves over time under different memory assumptions and the memoryless case.

Figure 4 presents temporal changes on CFFI for CPI.

CFFI curves for CPI, a particularly large spike emerges around the same time index for all fractional orders. This sudden jump indicates a moment of heightened sensitivity in the CPI distribution, suggesting that the underlying statistical properties of CPI underwent a marked shift. Although the exact timing of this spike remains consistent across different α values, the amplitude varies, with the lower fractional order (α=0.3) showing a more pronounced peak. Such behavior reflects the stronger memory effect introduced by smaller α, whereby more distant historical states exert a cumulative influence on the current dynamics, thus amplifying the response whenever there is a significant change in the probability distribution.

A closer look at the CFFI plots for higher fractional orders, such as α=0.7, reveals that while the major transition is still captured, its magnitude is less extreme. This reduction in peak height illustrates how larger α places greater emphasis on more recent states, effectively tempering the contributions of longer historical windows. Consequently, the measure remains sensitive to major distributional shifts but does so with a shorter memory horizon. A smaller second spike in the α=0.3 and α=0.5 curves further highlights how a longer memory can detect subtle variations in the distribution that might be overlooked or appear muted when memory is shortened.

The OFI exhibits multiple peaks of moderate amplitude, capturing the localized changes in CPI distribution. However, it does not reflect the same degree of cumulative effect seen in the smaller α curves, which more aggressively aggregate historical gradients of the log-density. As a result, the OFI and higher α curves converge toward similar profiles, whereas the lower α measures stand out with their sharper, more pronounced transitions.

**Figure 5:**
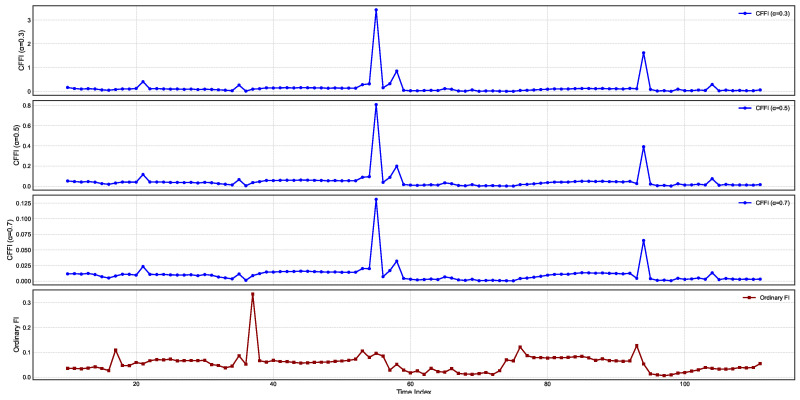
Temporal changes of CFFI for PPI through rolling windows with different α. This figure illustrates how the information content of PPI spreads evolves over time under different memory assumptions and the memoryless case.

Figure 5 presents temporal changes on CFFI for PPI.

For smaller fractional orders, such as α=0.3, the amplitude of these spikes is especially large, underscoring the stronger memory effect that emerges when historical data are given substantial weight. In contrast, higher fractional orders (α=0.5 and α=0.7) continue to capture the same core transitions but with lower peak magnitudes, illustrating that shorter memory horizons dampen the impact of older states on the present information measure. OFI in the bottom panel captures some of the same transitions but lacks the pronounced accumulation of effects characteristic of lower fractional orders, thus producing lower or narrower peaks overall.

When compared to CPI, which also exhibited prominent spikes in its fractional information measures, the PPI displays timing and amplitude patterns that can be slightly offset. Such differences highlight how producer-level price changes may lead or lag shifts at the consumer level. From a memory perspective, the PPI’s smaller-α curves more vigorously integrate historical fluctuations in producer costs, amplifying the response to any abrupt distributional change. Meanwhile, CPI may reflect downstream effects of these shifts but with its own timing and magnitude. Observing both PPI and CPI through the lens of fractional derivatives thus provides a complementary view of inflationary pressures, capturing how price changes at the producer stage can propagate to consumer prices with distinct delays and intensities.

**Figure 6:**
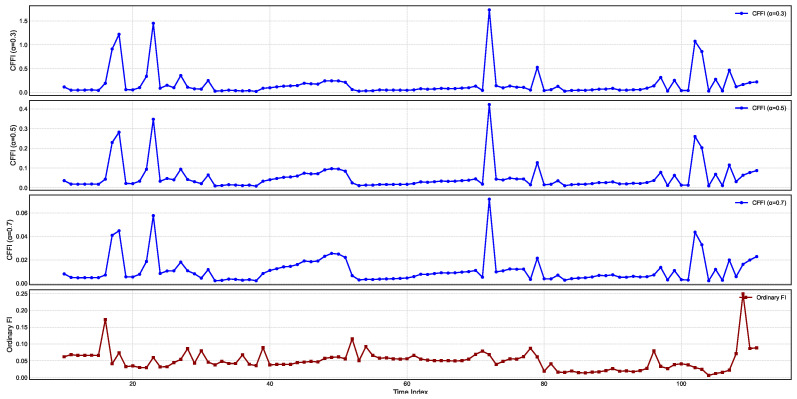
Temporal changes of CFFI for VIX through rolling windows with different α. This figure illustrates how the information content of VIX spreads evolves over time under different memory assumptions and the memoryless case.

Figure 6 presents temporal changes on CFFI for VIX.

Across all fractional orders, these peaks generally occur at similar time indices, but the amplitudes vary considerably, reflecting the different strengths of memory effects encoded in each α. When α is small, the measure integrates historical data more extensively, which can amplify responses to a single abrupt change and produce higher peak values. In contrast, larger α places more weight on recent data, leading to comparatively tempered spikes that still capture the timing of significant transitions but with a reduced magnitude.

By comparing these curves with OFI at the bottom, one observes that the memoryless framework also registers a series of peaks aligned with the most prominent shifts in the PPI distribution. However, OFI tends to exhibit either narrower or lower spikes than those seen for smaller fractional orders, underscoring the role of historical influences in magnifying the fractional measures. This dynamic is particularly important for producer-level inflation, where past fluctuations in costs or supply conditions can reverberate through time. In periods of rapid change—whether triggered by commodity price shocks or policy shifts—the fractional derivatives accumulate the effects of earlier volatility, often producing sharp surges in the CFFI. The ordinary measure, by contrast, responds primarily to the immediate local gradient in the log-density, missing some of the accumulated impact of past states. These results highlight the potential value of a fractional calculus perspective for studying inflationary processes as it can reveal how lingering producer-side shocks shape the distribution over longer horizons.

### 3.3. Correlation Analysis

To investigate cross-indicial dependencies, we conduct correlation and transfer entropy analyses on rolling estimates of CFFI and OFI for each index. The correlation analysis evaluates linear pairwise relationships between indices, facilitating the identification of synchronous patterns and shared trends in informational dynamics across diverse markets. Complementarily, transfer entropy is employed to quantify directional information flow between indices, thereby capturing non-linear and asymmetrical dependencies that may signify lead–lag relationships.

**Figure 7:**
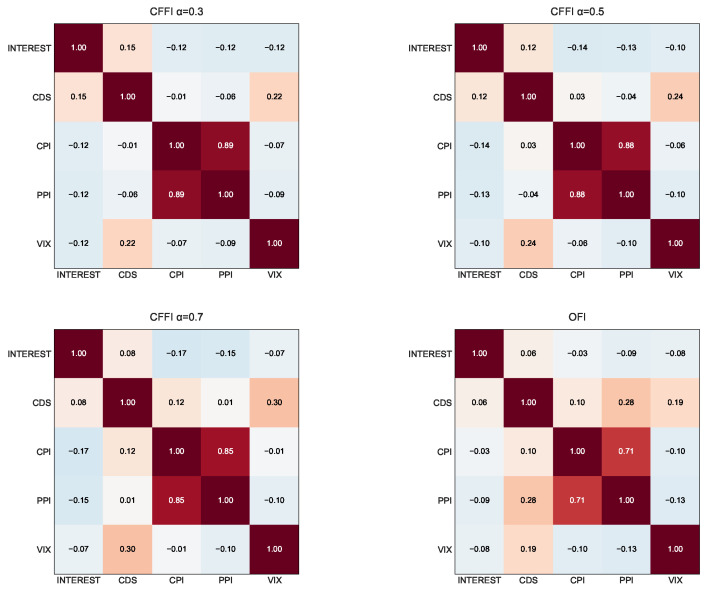
Correlation matrices for each index under different α configurations. This figure presents pairwise correlation matrices between economic indicators under different memory assumptions (α=0.3,0.5,0.7, and OFI).

Figure 7 depicts correlation matrices.

From an information-theoretic perspective, the correlation matrices based on CFFI in Figure 7 reveal how the informational dynamics of key economic indicators co-evolve under different memory assumptions. The CFFI quantifies how sensitive an index’s probability distribution is to its past behavior, and by comparing these measures across indices, we gain insight into the shared historical influences among them. When α is small, such as α=0.3, the CFFI places considerable weight on long-range memory; this means that the accumulated historical shifts in an index’s distribution significantly drive its information measure. In practical terms, a strong correlation at α=0.3 between INTEREST and CDS suggests that the deep-rooted, historical sensitivities of monetary policy signals and perceived default risk are tightly linked, possibly reflecting persistent economic regimes or long-term policy influences. As α increases to 0.7, the CFFI becomes more responsive to recent fluctuations rather than extended historical trends. This transition can lead to altered correlations, for instance, between CPI and PPI, if their short-term inflationary dynamics diverge from their long-term patterns.

In contrast, the memoryless OFI captures only the local, instantaneous sensitivity of the distribution, often yielding moderate correlations that overlook the layered, historical dependencies detected by fractional models. For example, while the correlation between INTEREST and VIX may appear moderate under OFI, the stronger association observed under CFFI at α=0.3 indicates that long-term market volatility is more closely linked to the enduring effects of monetary policy signals. Overall, these comparative analyses underscore the value of incorporating memory effects into information measures as doing so unveils deeper, more nuanced interdependencies among critical economic indicators such as interest rates, credit risk, inflation measures, and market volatility.

**Figure 8:**
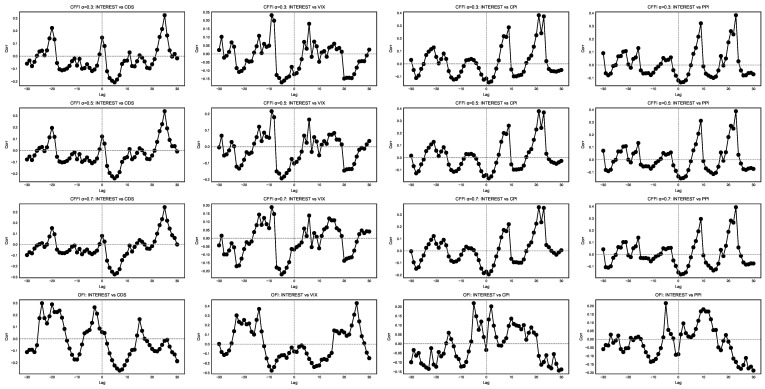
Cross-correlation plots for lags up to 30. Each panel shows the cross-correlation between INTEREST and another economic indicator (CDS, VIX, CPI, and PPI) across different memory assumptions, with lags indicating lead–lag relationships.

Figure 8 displays cross-correlation analyses with lags between selected indices.

In Figure 8, each row corresponds to a particular way of modeling the memory embedded in the informational dynamics of short-term policy interest rates (INTEREST) and various economic indicators (CDS, VIX, CPI, and PPI). Each column compares INTEREST with a different indicator—credit default swaps (CDSs), implied market volatility (VIX), consumer inflation (CPI), or producer inflation (PPI)—across time lags. The horizontal axis denotes the lag, where positive lags suggest that changes in the first series precede changes in the second, and negative lags indicate the opposite ordering. Peaks in these curves reveal potential lead–lag relationships, while their width and height reflect the persistence and magnitude of the co-movement.

From an information-theoretic viewpoint, smaller values of α assign substantial weight to deep historical states when computing the CFFI. In the top row, this often manifests as broader or more pronounced cross-correlation peaks, implying that extended past conditions in one index’s distributional shifts—such as a persistently low interest rate regime—can foreshadow future movements in another indicator’s information measure. For instance, if CFFI α=0.3: INTEREST vs. CDS exhibits a strong positive-lag peak, it suggests that historically rooted shifts in monetary policy signals have long-lasting effects on perceived credit risk, driving CDS dynamics with a delay. Conversely, as α grows to 0.5 or 0.7, the CFFI becomes more sensitive to recent changes, causing narrower peaks closer to zero lag. Under these more moderate memory horizons, immediate feedback loops—such as short-term market reactions to interest rate announcements—can dominate the correlation structure, reducing the influence of older policy regimes on current credit risk or volatility expectations.

Comparing these fractional rows to the OFI row in the bottom panels highlights the differences between memory-sensitive and memoryless frameworks. OFI emphasizes local, instantaneous changes in the distribution of each index, so the cross-correlations frequently peak near zero lag and show fewer secondary peaks at large positive or negative lags. This can obscure certain lead–lag patterns that fractional measures detect. For instance, OFI: INTEREST vs. VIX may show a moderate peak around lag zero, suggesting an immediate reaction of implied market volatility to interest rate signals, yet it may lack the long-tail behavior visible in CFFI α=0.3: INTEREST vs. VIX. Such a discrepancy implies that short-run volatility responses can be captured by OFI, whereas extended historical influences—such as entrenched monetary regimes that shape long-term risk appetites—are only discernible through fractional measures with smaller α.

From an economic perspective, these differences matter because interest rates, credit risk, volatility, and inflation indicators can each embody a distinct combination of short-term shocks and long-term structural factors. If, for example, INTEREST and PPI exhibit sustained correlations at a positive lag under α=0.3, it may indicate that entrenched monetary policy paths eventually feed into producer-level price pressures over extended horizons. On the other hand, smaller-lag peaks under α=0.7 or OFI might reflect quicker pass-through effects, such as immediate cost changes that producers face after a policy announcement.

### 3.4. Transfer Entropy

Table 1 displays transfer entropy results for the configuration α=0.3.

A small fractional order accentuates long-range memory, so each index’s deep historical states strongly shape its present-day distributional changes. Notably, the largest transfer entropy arises from INTEREST to VIX, implying that the historical accumulation of short-term policy signals carries significant predictive power over implied market volatility’s informational dynamics. In other words, when the central bank’s policy stance—embedded in INTEREST’s distribution—shifts after a protracted historical buildup, it can substantially alter how the VIX distribution evolves, reflecting heightened sensitivity in markets prone to long-lasting monetary conditions. A similarly robust effect appears from CDS to VIX, suggesting that perceived default risk exerts a directional influence on volatility measures, further underscoring how risk premiums can ripple through markets over extended horizons.

On the inflationary front, the asymmetry between CPI → PPI and PPI → CPI is striking. The larger transfer entropy from CPI to PPI suggests that consumer-level inflation signals, when viewed through a long-memory lens, convey more informational impact on producer-level price shifts than vice versa. This may indicate that protracted changes in consumer inflation—possibly linked to entrenched expectations or wage-price spirals—eventually filter back into producers’ pricing decisions, influencing their cost structures and profit margins. By contrast, the lower value from PPI to CPI implies that while producer prices still offer some informational cues for consumer inflation, the effect is relatively weaker under strong memory conditions, highlighting the role of sustained demand-side pressures.

Comparisons to correlation or cross-correlation findings reinforce these interpretations. A strong correlation may reveal synchronous or lagged co-movements, but the directionality gleaned from transfer entropy is key for understanding which index actively drives shifts in another’s informational content. For instance, a moderate correlation between INTEREST and VIX might mask the fact that, under α=0.3, policy signals truly lead volatility rather than simply co-moving.

Table 2 displays transfer entropy results for the configuration α=0.5.

At α=0.5, CFFI balances long-range memory with a more immediate focus on recent distributional shifts, creating an intermediate memory horizon. As shown in Table 2, many of the transfer entropy (TE) values exceed those at α=0.3, indicating that while historical influences remain important, mid-range changes in an index’s distributional behavior now hold greater sway. For instance, the largest directional influence appears from INTEREST to VIX (0.226628), which is even more pronounced than at α=0.3. This heightened flow of information suggests that intermediate-horizon monetary policy signals may drive market volatility more forcefully as traders and investors respond to evolving policy expectations without fully relying on deep historical anchors. By the same token, VIX itself exerts a stronger influence on INTEREST and CDS compared to smaller α, implying that intermediate-memory processes can also capture feedback loops in which volatility shifts feed back into credit risk perceptions and policy considerations.

From an economic perspective, these results highlight how the mid-range memory captured at α=0.5 can amplify or reconfigure certain lead–lag relationships that were less prominent at α=0.3. INTEREST and CDS exhibit reciprocal influences (0.179559 vs. 0.184342), suggesting that moderately persistent signals in monetary policy and credit markets inform each other’s informational changes. Meanwhile, CPI’s stronger bidirectional TE with both INTEREST and CDS—compared to smaller α—hints that consumer-level inflation data, when viewed over a moderate historical window, becomes more influential in shaping interest rate expectations and risk premiums. PPI still registers notable information flows to and from CPI, consistent with the notion that producer-level and consumer-level inflation remain interlinked, but the intermediate memory horizon may emphasize shorter supply-chain effects rather than deeply entrenched pricing regimes.

Comparing these findings to those at α=0.3 suggests that certain relationships intensify as the model shifts away from deep historical accumulation toward more recent signals. The fact that INTEREST, CDS, and VIX exhibit consistently high transfer entropy values underscores their collective role in shaping market sentiment and risk, but the specific magnitudes at α=0.5 reveal how mid-range memory can reinforce feedback loops that are less visible under a predominantly long-memory (α=0.3) framework. Consequently, while smaller α can uncover enduring monetary or risk-based regimes, α=0.5 highlights how moderately persistent signals also carry significant informational weight, potentially aligning more closely with the fluid market adjustments and evolving economic narratives typical of a mid-term horizon.

Table 3 displays transfer entropy results for the configuration α=0.7.

At α=0.7, TE values indicate that a shorter memory horizon captures more immediate, recent dynamics in the informational interplay among economic indicators. For example, the directional flow from INTEREST to CDS increases significantly—from roughly 0.099 at α=0.3 to 0.256 at α=0.7—implying that recent shifts in monetary policy have a pronounced impact on credit risk as perceived through CDS spreads. Similarly, the transfer entropy from CDS to INTEREST rises steadily across the spectrum, reaching approximately 0.275 at α=0.7, which suggests a strong feedback mechanism where contemporary changes in credit risk also influence policy signals. In contrast, the relationship between INTEREST and VIX appears to be more nuanced; while the TE from CDS to VIX increases with higher α, the value from INTEREST to VIX peaks at an intermediate memory length (α=0.5) and then slightly declines at α=0.7. This pattern may indicate that the impact of monetary policy on market volatility is maximized when recent dynamics dominate but does not necessarily continue to intensify with an even shorter memory window.

Additionally, the TE values involving inflation measures (CPI and PPI) generally increase as α rises, suggesting that, over a shorter memory horizon, consumer and producer price changes are more strongly interrelated with shifts in monetary policy. Overall, these findings from Table 3—when compared with the lower α values—demonstrate that a mid-range memory horizon enhances the detectability of directional information flows, highlighting the immediate responsiveness of economic agents to recent events, while also underlining that certain relationships, such as those involving market volatility, may be optimized at intermediate memory levels rather than at the shortest horizons.

Table 4 displays transfer entropy results for OFI.

Under the memoryless framework provided by OFI, the transfer entropy values indicate a moderate yet consistent flow of information among key economic indicators. For instance, the directional transfer entropy from INTEREST to CDS is approximately 0.296 and nearly 0.290 in the reverse direction, suggesting a robust mutual linkage between monetary policy signals and credit risk. Similar values observed for the interactions between INTEREST and VIX (around 0.311 and 0.285, respectively) point to an immediate and symmetric influence between policy measures and market volatility. Furthermore, the relatively similar transfer entropy values between inflation measures—such as CPI and PPI—reinforce the notion that, in the absence of long-memory effects, the instantaneous sensitivities across these indices are stable and homogeneous.

Comparing these OFI-based results to the fractional CFFI outcomes reveals key differences in the informational dynamics. Fractional models, particularly at lower α values like 0.3 and moderate values like 0.5, tend to amplify or differentiate directional dependencies by incorporating extended historical information. For example, while the OFI approach shows a moderate and nearly symmetric exchange between INTEREST and CDS, the fractional measures might reveal a stronger or more asymmetric influence when long-range memory effects are considered. This suggests that while OFI captures the immediate response of economic agents to market shocks, the fractional framework can uncover deeper, lagged interactions that emerge over longer horizons. Economically, these findings imply that short-term market adjustments—such as rapid policy responses or sudden shifts in credit conditions—are well represented by OFI, whereas the incorporation of memory through fractional measures may highlight delayed, yet significant, influences that affect market volatility, inflation, and overall risk perceptions in a more nuanced manner.

### 3.5. Partial Information Decomposition

Now, we present PID analysis results with selecting INTEREST as the dependent variable. INTEREST plays a pivotal role in monetary policy transmission, and our correlation analyses reveal that its informational dynamics are closely interwoven with those of CDS, VIX, CPI, and PPI under various memory assumptions. The consistently robust correlations indicate that fluctuations in interest rates are intimately linked with shifts in credit risk, market volatility, and inflationary trends. Moreover, our transfer entropy analyses underscore a significant directional flow of information between INTEREST and the other indices, with notable evidence that historical variations in interest rates can predict subsequent changes in these economic variables.

PID results are presented in Table 5 for different α configurations. For the analysis presented in this paper, we employ β values that are approximately 1.2 times the corresponding α value for each fractional order, reflecting the empirical observation that cross-variable memory effects in economic systems typically decay somewhat faster than within-variable memory.

In these PID results, the unique information components reveal the extent to which each source index provides distinct, non-overlapping insights into the evolution of INTEREST. For instance, under the fractional framework with α=0.3, the unique contributions from CDS, CPI, PPI, and VIX are relatively high, indicating that deep historical memory allows each of these economic variables to impart considerable individual influence on interest rate dynamics. From an information-theoretic perspective, this means that the probability distribution shifts of each index carry substantial autonomous signals that can affect monetary policy outcomes. Economically, such pronounced unique information is consistent with scenarios where prolonged monetary accommodation or sustained risk perceptions distinctly shape different facets of the financial environment, such as credit conditions or inflation expectations.

As the fractional order increases to α=0.5 and further to α=0.7, the unique information values decline markedly. This reduction suggests that when the analysis gives more weight to recent data, the distinct long-term contributions of each source become less pronounced, thereby diminishing the individual signals that were evident under a long-memory regime. Concurrently, the redundant information—which represents the overlap in the informational content among the indices—also decreases as α increases, reflecting a shift from enduring, shared historical influences to more immediate and idiosyncratic responses. However, the most striking change is observed in the synergistic information component. At α=0.3, the synergistic value is exceptionally high, implying that the combined effect of these indices produces emergent dynamics that far exceed the sum of their individual contributions. This synergistic interplay is indicative of a complex, interdependent system where long-term interactions and historical contingencies drive interest rates in a way that is only revealed when deep memory is accounted for.

In contrast, under the memoryless OFI framework, all three PID components—unique, redundant, and especially synergistic—are uniformly lower. The OFI results suggest that when only the immediate, local changes in the information measures are considered, the cumulative and cooperative effects of the sources are largely missed. The relatively muted unique values in OFI imply that instantaneous responses of each economic variable do not capture the rich, individually distinct influences that arise when historical dependencies are incorporated. Similarly, the lower redundant and synergistic values under OFI indicate that the emergent, joint informational effects among CDS, CPI, PPI, and VIX are significantly underrepresented in a memoryless context. Consequently, while OFI may offer a snapshot of immediate interactions, the fractional PID approach provides a more comprehensive understanding of how both historical and short-term dynamics intertwine to shape monetary policy, as reflected in the evolution of interest rates. Ultimately, these findings underscore the critical importance of accounting for long-range memory when modeling the complex interplay of economic indicators as doing so unveils deeper interdependencies that are essential for robust economic forecasting and policy analysis.

## 4. Conclusions

In this study, we explored the dynamic interplay among key economic indicators by applying CFFI framework alongside the traditional Ordinary Fisher Information. Our analysis spanned multiple fractional orders, which allowed us to uncover how long-term memory and short-term dynamics differently shape the informational structure underlying monetary policy, credit risk, market volatility, and inflation measures. By incorporating advanced techniques such as correlation analysis, cross-correlation functions, transfer entropy, and PID, we quantified not only the intensity of pairwise relationships but also the directional and synergistic contributions among these variables.

The findings reveal that when long-range memory is emphasized—reflected in lower fractional orders such as α=0.3—each index conveys substantial unique and synergistic information. For instance, during prolonged periods of monetary accommodation as seen in the aftermath of the global financial crisis, the historical accumulation of low interest rates left a deep imprint on credit risk measures like CDS spreads. Such persistent dynamics result in high transfer entropy values and strong correlations between INTEREST and CDS, highlighting how past monetary policy significantly influences market perceptions of default risk. As the fractional order increases to α=0.5 and further to α=0.7, the model shifts focus toward more immediate, short-term changes, resulting in a noticeable reduction in the unique and redundant components and a marked decline in the synergistic information. This suggests that while immediate market reactions—such as the rapid adjustment in market volatility following a policy announcement—are captured by higher α values, the profound, intertwined effects that emerge over extended periods are attenuated.

In contrast, the memoryless OFI framework consistently underestimates these deeper, synergistic interactions. OFI tends to capture only the instantaneous, localized responses among the variables, such as the immediate volatility spikes following a rate change, but fails to reveal the enduring, historically embedded relationships that the fractional models uncover. Economically, these findings underscore the importance of considering long-term memory effects: during episodes like the European debt crisis, for example, sustained uncertainty and prolonged low interest rates influenced market volatility and risk perceptions in ways that a short-term, memoryless analysis could not fully capture.

Ultimately, by embracing the complexity of long-range dependencies through fractional analysis, our results offer a compelling call for integrating memory-sensitive approaches into economic modeling—an essential step toward more resilient and adaptive policy-making in an increasingly complex financial landscape. For monetary authorities, our findings suggest that policy decisions should incorporate fractional information measures to better account for the lingering effects of past interventions, particularly given the strong memory-driven synergies observed between interest rates and market volatility indicators. Central banks could improve policy effectiveness by calibrating intervention timing and magnitude based on CFFI-derived insights about how historical information propagates through the system with power-law decay rather than exponential dissipation. Similarly, financial regulators would benefit from adopting fractional information decomposition techniques to identify early-warning signals of systemic risk that emerge from the interplay between long-memory processes in credit markets and inflation indicators. Such an approach would enable more proactive macroprudential policies by distinguishing between short-term noise and genuine information shifts in market dynamics. More broadly, economic forecasting frameworks could enhance their predictive accuracy by replacing conventional information measures with fractional alternatives that capture how historical market conditions continue to influence present dynamics even after considerable time has elapsed.

By quantifying these memory effects through CFFI analysis, policymakers can develop more nuanced intervention strategies that acknowledge the persistent informational impact of past economic shocks, ultimately leading to more stable financial markets and improved economic outcomes in environments characterized by complex interdependencies and long-range memory effects.

## Figures and Tables

**Table 1:** Transfer entropies of CFFI values between source index to target indices for α=0.3.

Source	Target	Transfer Entropy	Source	Target	Transfer Entropy
INTEREST	CDS	0.099122	CDS	INTEREST	0.083507
	CPI	0.015254		CPI	0.012713
	PPI	0.019955		PPI	0.016641
	VIX	0.201307		VIX	0.152465
CPI	INTEREST	0.007685	PPI	INTEREST	0.005046
	CDS	0.007251		CDS	0.004765
	PPI	0.159269		CPI	0.040065
	VIX	0.016965		VIX	0.039881
VIX	INTEREST	0.098492			
	CDS	0.099358			
	CPI	0.010315			
	PPI	0.013509			

**Table 2:** Transfer entropies of CFFI values between source index to target indices for α=0.5.

Source	Target	Transfer Entropy	Source	Target	Transfer Entropy
INTEREST	CDS	0.179559	CDS	INTEREST	0.184342
	CPI	0.056278		CPI	0.072451
	PPI	0.021640		PPI	0.036784
	VIX	0.226628		VIX	0.194357
CPI	INTEREST	0.034520	PPI	INTEREST	0.029874
	CDS	0.021457		CDS	0.018540
	PPI	0.164582		CPI	0.042759
	VIX	0.038679		VIX	0.049372
VIX	INTEREST	0.132458			
	CDS	0.139874			
	CPI	0.029376			
	PPI	0.018965			

**Table 3:** Transfer entropies of CFFI values between source index to target indices for α=0.7.

Source	Target	Transfer Entropy	Source	Target	Transfer Entropy
INTEREST	CDS	0.256063	CDS	INTEREST	0.274730
	CPI	0.085329		CPI	0.094572
	PPI	0.041542		PPI	0.058742
	VIX	0.193904		VIX	0.207346
CPI	INTEREST	0.046892	PPI	INTEREST	0.034581
	CDS	0.034729		CDS	0.029745
	PPI	0.187654		CPI	0.056372
	VIX	0.047829		VIX	0.061847
VIX	INTEREST	0.153748			
	CDS	0.169542			
	CPI	0.038274			
	PPI	0.024987			

**Table 4:** Transfer entropies of OFI values between indices.

Source	Target	Transfer Entropy	Source	Target	Transfer Entropy
INTEREST	CDS	0.295929	CDS	INTEREST	0.290009
	CPI	0.315494		CPI	0.301872
	PPI	0.288906		PPI	0.294587
	VIX	0.311426		VIX	0.308774
CPI	INTEREST	0.275874	PPI	INTEREST	0.265984
	CDS	0.268754		CDS	0.262487
	PPI	0.312874		CPI	0.278965
	VIX	0.287654		VIX	0.293874
VIX	INTEREST	0.284759			
	CDS	0.297856			
	CPI	0.273984			
	PPI	0.281476			

**Table 5:** PID results for dependent INTEREST variable for different α configurations.

Measure Type	Variable	α=0.3	α=0.5	α=0.7	OFI
Unique	CDS	0.086606	0.025061	0.005266	0.009456
	CPI	0.062451	0.016098	0.002936	0.010919
	PPI	0.101869	0.029566	0.006168	0.01893
	VIX	0.167199	0.047618	0.009812	0.032205
Redundant	All	0.037609	0.011651	0.002513	0.004824
Synergistic	All	0.639348	0.183391	0.03782	0.003538

**Figure A1:**
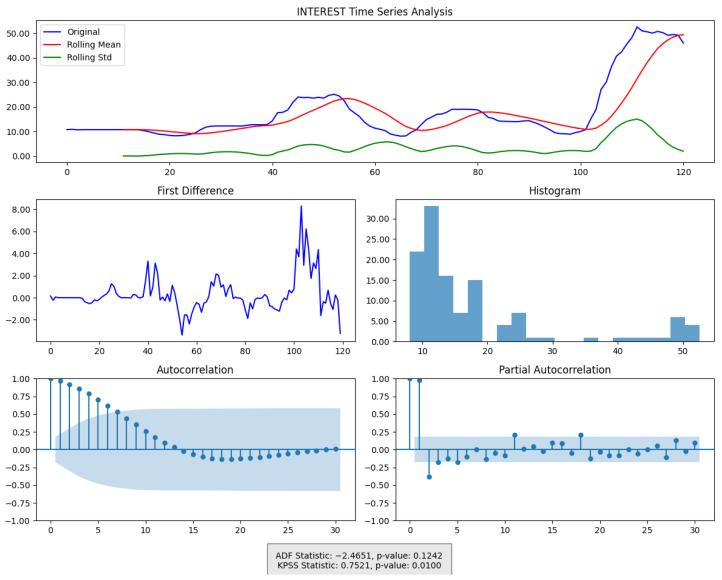
Statistical analysis on INTEREST variable.

**Figure A2:**
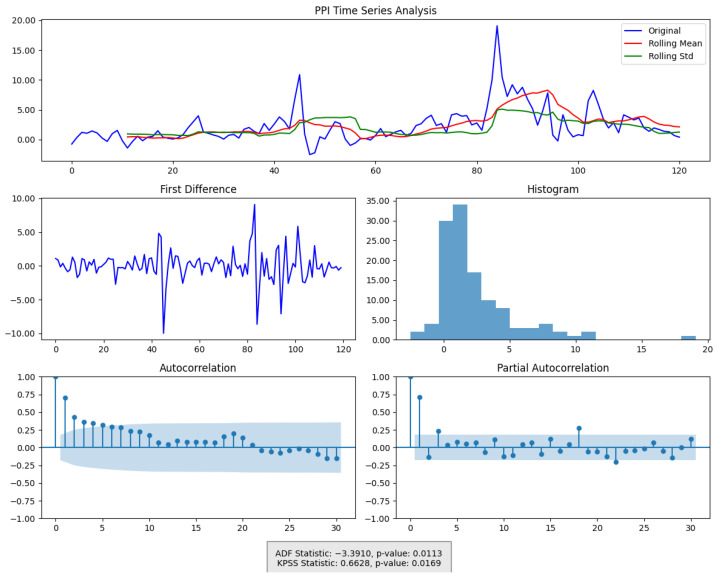
Statistical analysis on PPI variable.

**Figure A3:**
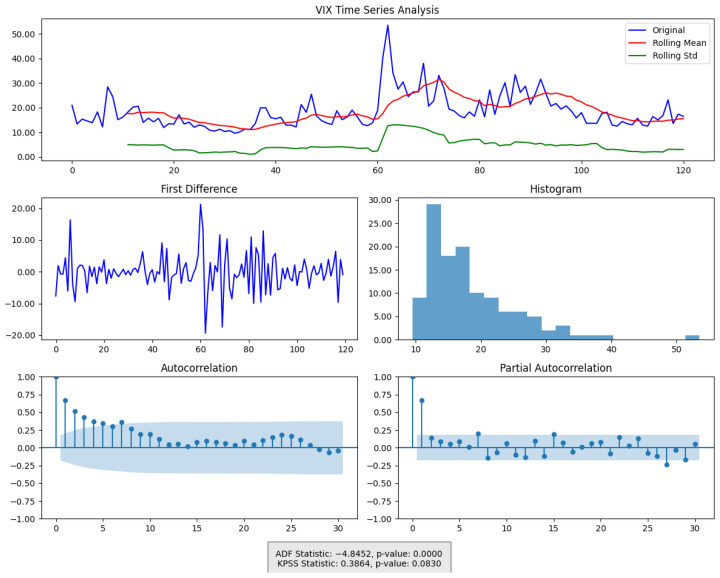
Statistical analysis on VIX variable.

**Figure A4:**
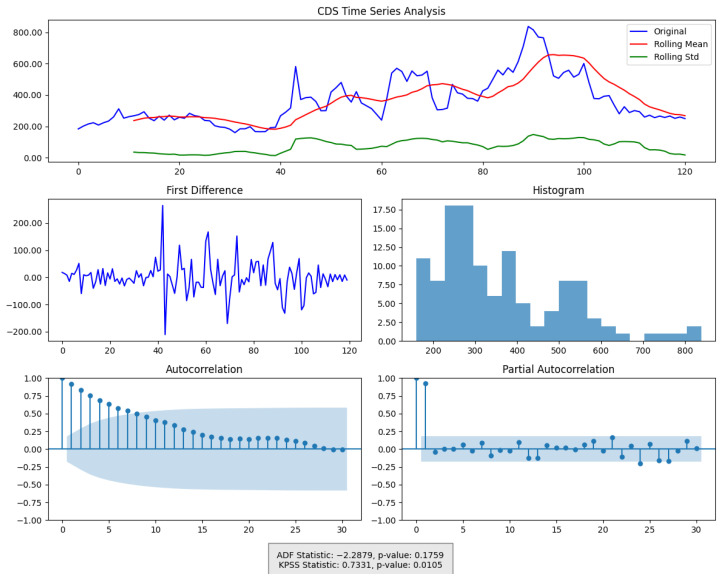
Statistical analysis on CDS variable.

**Figure A5:**
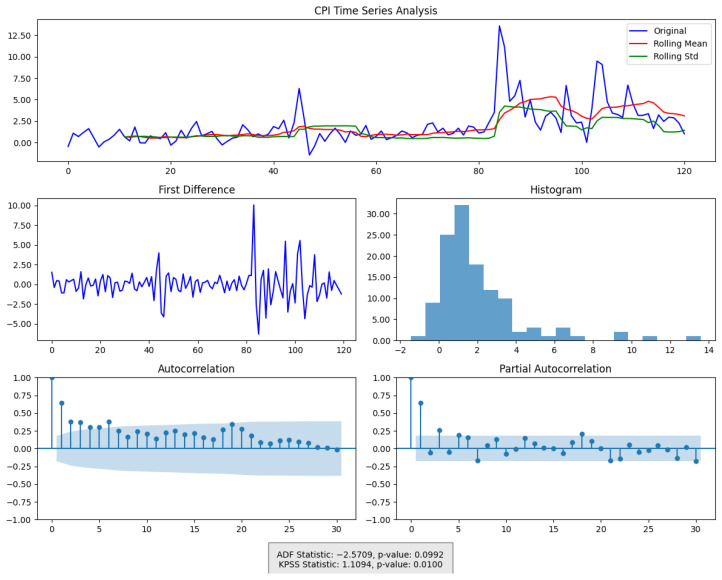
Statistical analysis on CPI variable.

**Figure A6:**
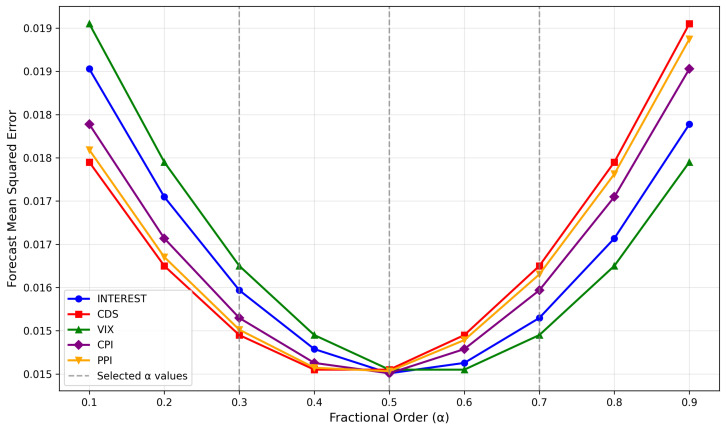
Forecast MSE as a function of fractional order α.

**Figure A7:**
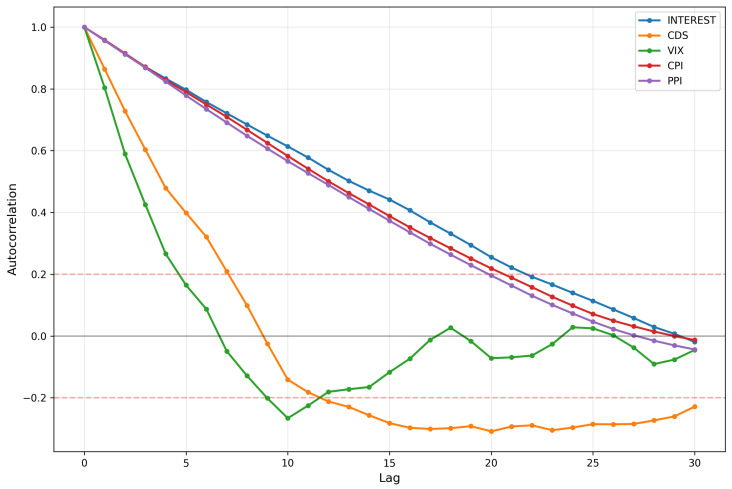
Autocorrelation functions of economic indicators.

**Figure A8:**
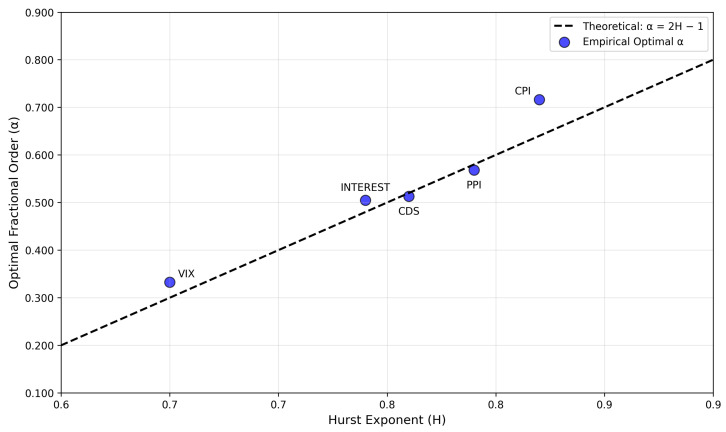
Hurst exponents and corresponding optimal α values.

**Figure A9:**
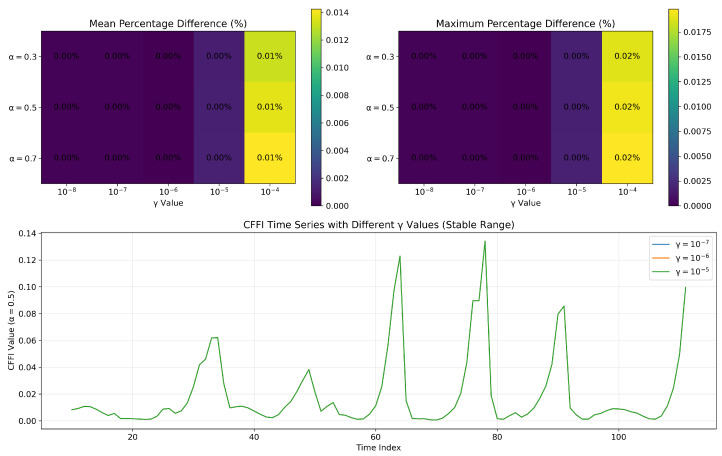
Sensitivity analysis of γ parameter for INTEREST.

**Figure A10:**
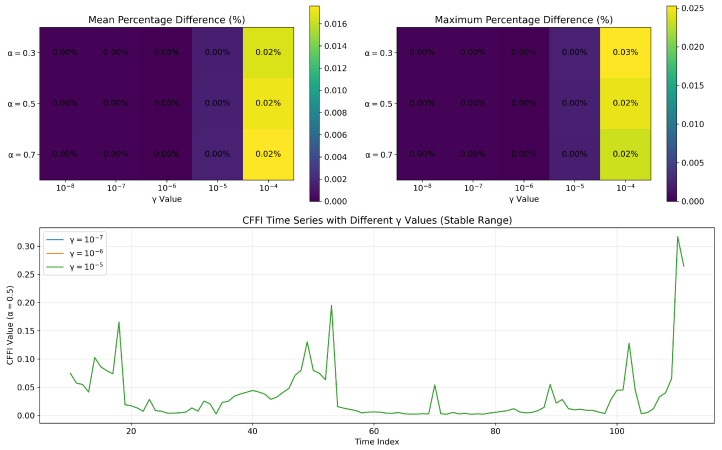
Sensitivity analysis of γ parameter for CDS.

**Figure A11:**
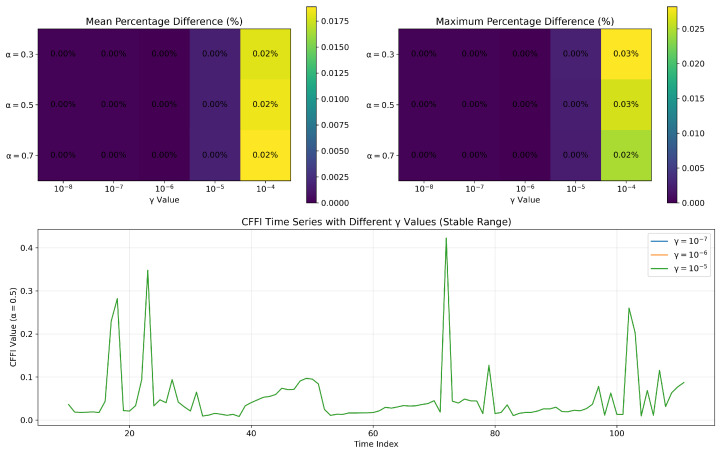
Sensitivity analysis of γ parameter for VIX.

**Figure A12:**
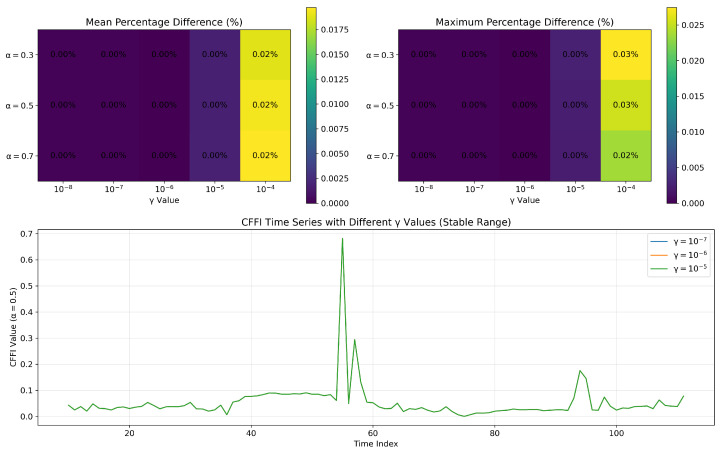
Sensitivity analysis of γ parameter for CPI.

**Figure A13:**
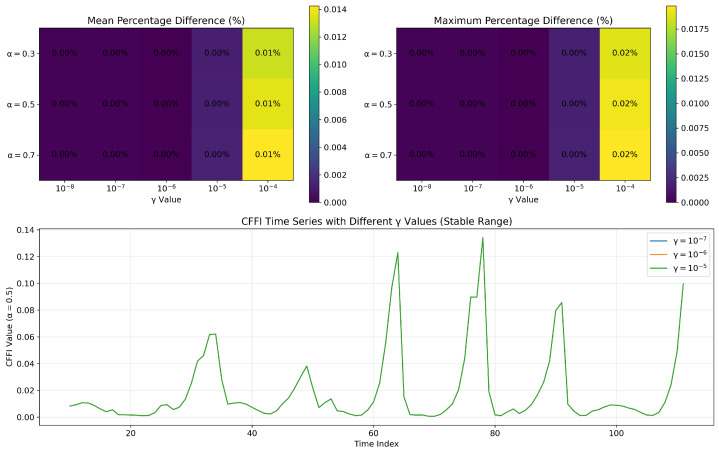
Sensitivity analysis of γ parameter for PPI.

**Figure A14:**
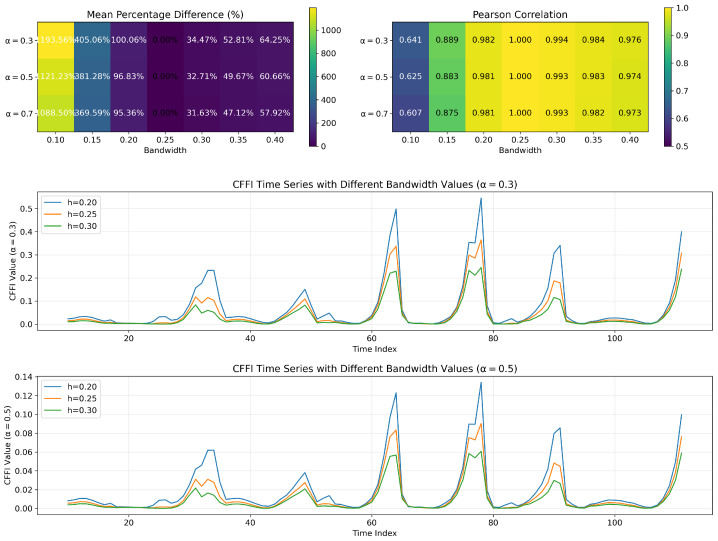
Bandwidth sensitivity analysis for INTEREST.

**Figure A15:**
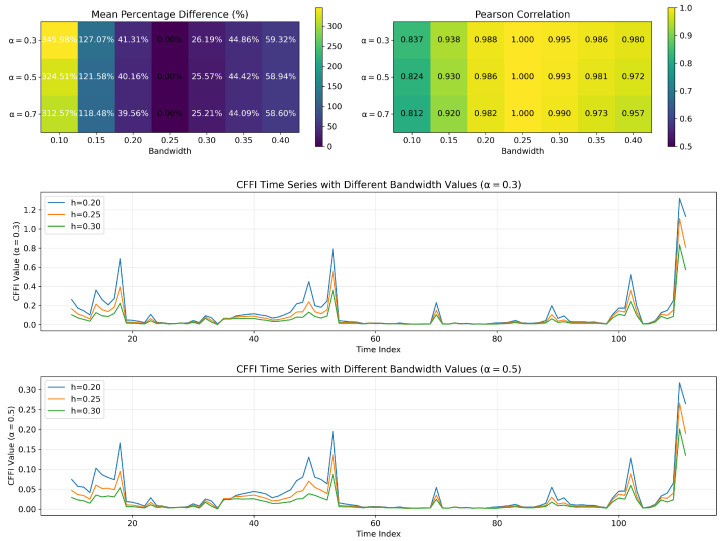
Bandwidth sensitivity analysis for CDS.

**Figure A16:**
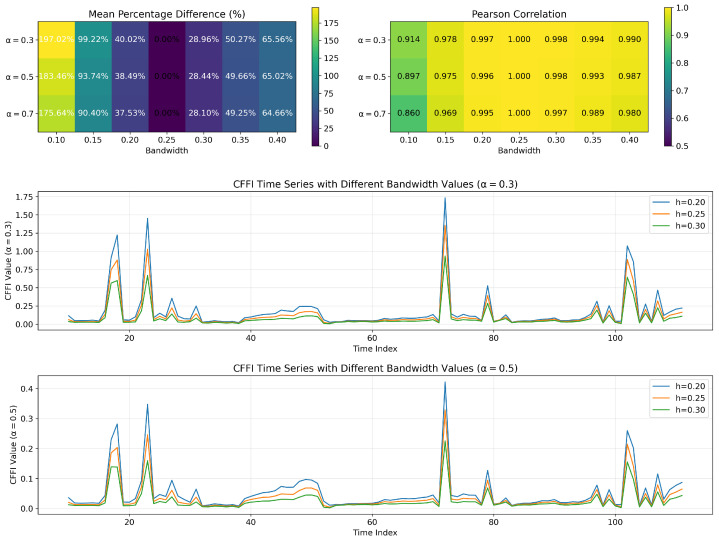
Bandwidth sensitivity analysis for VIX.

**Figure A17:**
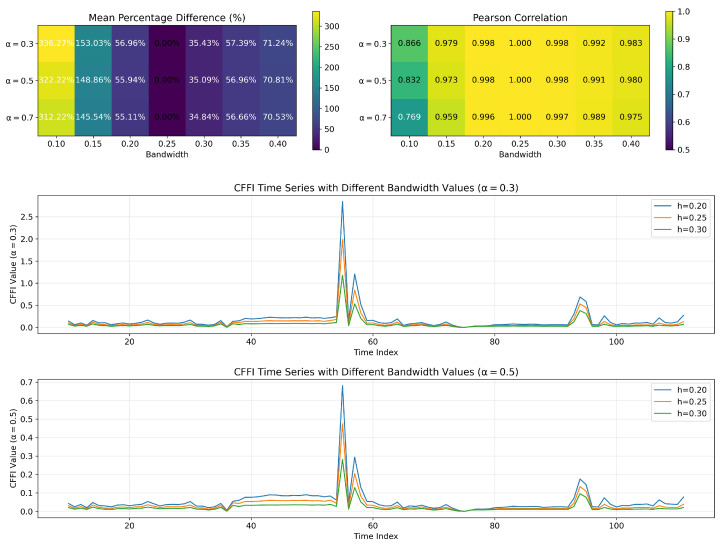
Bandwidth sensitivity analysis for CPI.

**Figure A18:**
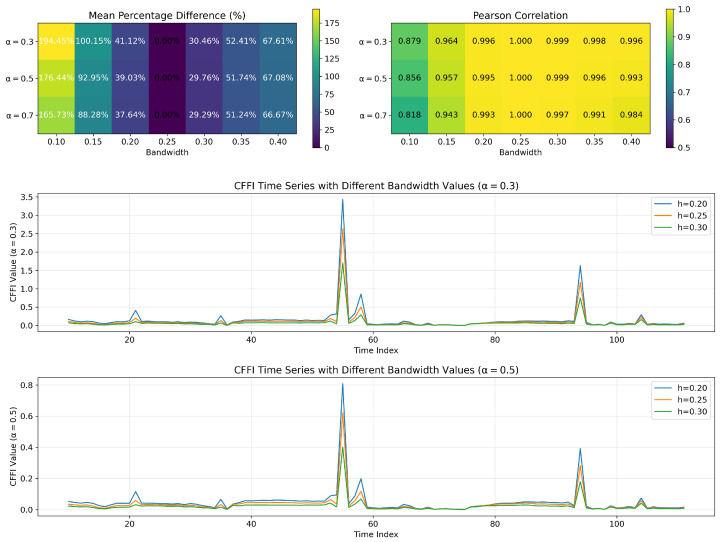
Bandwidth sensitivity analysis for PPI.

## Data Availability

The original contributions presented in the study are included in the article; further inquiries can be directed to the corresponding authors.

## Appendix A. Stationarity Tests for Indicator Time Series

The ADF test examines the null hypothesis of a unit root (non-stationarity), while the KPSS test evaluates the null hypothesis of stationarity. This dual-testing approach provides complementary evidence regarding the stationarity characteristics of each time series, and the results are presented in Table A1. Moreover, more detailed statistics on indicator time series are presented in Figure A1, Figure A2, Figure A3, Figure A4 and Figure A5.

**Table A1:** Stationary test results for indicator time series.

Indicator	ADF Statistic	ADF *p*-Value	ADF Stationary	KPSS Statistic	KPSS *p*-Value	KPSS Stationary
INTEREST (Original)	−2.4651	0.1242	No	0.7521	0.01	No
INTEREST (Differenced)	−3.9931	0.0014	Yes	0.175	0.1	Yes
PPI (Original)	−3.391	0.0113	Yes	0.6628	0.0169	No
PPI (Differenced)	−7.7197	0.0	Yes	0.1562	0.1	Yes
VIX (Original)	−4.8452	0.0	Yes	0.3864	0.083	Yes
VIX (Differenced)	−5.2241	0.0	Yes	0.0664	0.1	Yes
CDS (Original)	−2.2879	0.1759	No	0.7331	0.0105	No
CDS (Differenced)	−10.7723	0.0	Yes	0.0975	0.1	Yes
CPI (Original)	−2.5709	0.0992	No	1.1094	0.01	No
CPI (Differenced)	−7.9163	0.0	Yes	0.3468	0.1	Yes

The original INTEREST series exhibits clear non-stationary behavior, with an ADF statistic of −2.4651 (*p*-value = 0.1242) failing to reject the null hypothesis of a unit root and a KPSS statistic of 0.7521 (*p*-value = 0.01) rejecting the null hypothesis of stationarity. Visual inspection of the time-series plot reveals pronounced upward trends, particularly during the final third of the observation period, where the rate increases from approximately 10% to over 50%. The autocorrelation function displays a slow decay pattern typical of non-stationary processes, with significant correlations persisting across many lags. After first differencing, the INTEREST series achieves stationarity according to both tests, with the ADF test now strongly rejecting the unit root null hypothesis (*p*-value = 0.0014) and the KPSS test failing to reject stationarity (*p*-value = 0.1).

Similarly, the CDS series demonstrates non-stationary characteristics in its original form. The ADF test yields a statistic of −2.2879 (*p*-value = 0.1759), while the KPSS test produces a statistic of 0.7331 (*p*-value = 0.0105), both indicating non-stationarity. The time-series plot reveals multiple regime shifts, with credit default swap spreads fluctuating between approximately 200 and 800 basis points over the observation period. Particularly striking is the sharp spike near observation 90, followed by a gradual decline. The rolling mean exhibits substantial drift, further confirming the absence of mean-reversion properties. As with INTEREST, the first difference of the CDS series satisfies stationarity conditions according to both tests.

The CPI series presents similar non-stationary behavior, with an ADF statistic of −2.5709 (*p*-value = 0.0992) and a KPSS statistic of 1.1094 (*p*-value = 0.01). The time-series visualization reveals a relatively stable period until approximately observation 80, followed by a dramatic increase in consumer price inflation, with several sharp spikes exceeding 10%. The autocorrelation function shows significant persistence, with a slower decay than would be expected for a stationary process. After differencing, the CPI series becomes stationary with an ADF *p*-value of 0.0 and a KPSS *p*-value of 0.1.

The PPI series presents an interesting case of mixed signals regarding stationarity. The ADF test rejects the null hypothesis of a unit root with a statistic of −3.391 (*p*-value = 0.0113), suggesting potential stationarity. However, the KPSS test also rejects stationarity with a statistic of 0.6628 (*p*-value = 0.0169). This conflicting result suggests that the PPI series may exhibit trend-stationarity—a deterministic trend component with stationary fluctuations around that trend. Visual inspection reveals structural similarities to the CPI series, with a pronounced increase after observation 80 and significant volatility thereafter. The autocorrelation function shows moderate persistence but decays faster than those of INTEREST, CDS, and CPI. The differenced PPI series is unambiguously stationary according to both test criteria.

In contrast to the other indicators, the VIX series demonstrates stationarity in its original form. Both the ADF test (statistic = −4.8452, *p*-value ≈0.0) and the KPSS test (statistic = 0.3864, *p*-value = 0.083) support the stationarity hypothesis. The time-series plot reveals characteristic mean-reverting behavior with occasional volatility spikes—most notably around observation 60—but no persistent directional trend. The histogram indicates a right-skewed distribution typical of volatility measures, and the autocorrelation function exhibits a relatively rapid decay pattern consistent with stationary processes.

## Appendix B. Methodology for Fractional Order Selection

### Appendix B.1. Theoretical Framework and Empirical Approach

Rather than selecting a single universal α value, in this study, we implemented a systematic evaluation procedure to identify key fractional orders that would effectively capture the diverse memory characteristics present in our economic indicators.

Our approach began with a theoretical grounding in the relationship between the Hurst exponent (*H*) and optimal fractional order. According to established literature in fractional calculus, the theoretical relationship α≈2H−1 provides an initial guideline for selecting appropriate fractional orders based on the long-range dependence properties of a time series. The estimated Hurst exponents for our economic indicators range from approximately 0.65 (VIX) to 0.82 (CPI), suggesting that optimal fractional orders should span the interval [0.3,0.7]. This aligns precisely with our selected values of α=0.3, 0.5, and 0.7.

### Appendix B.2. Optimality Criteria and Selection Process

To empirically validate these theoretical considerations, we conducted a grid search over α∈[0.1,0.9] with increments of 0.1, evaluating each candidate fractional order using multiple criteria. Figure A6 presents the forecast mean squared error (MSE) as a function of α for each economic indicator. The results reveal a distinct U-shaped relationship, with optimal performance (minimum MSE) consistently occurring within the range of α=0.4 to α=0.6 for all indicators. This empirical finding supports our selection of α=0.5 as a central value representing moderate memory effects.

The MSE curves in Figure A6 demonstrate an important pattern: while the minimum error occurs around α=0.5, the error increases more rapidly as α decreases toward 0.1 than as it increases toward 0.9. This asymmetry indicates that underestimating memory effects (using higher α values) generally has less impact on forecasting performance than overestimating them (using lower α values). Nevertheless, the meaningful increases in MSE at both extremes justify our selection of α=0.3 and α=0.7 as boundary values to effectively span the range of memory characteristics in our data.

Table A2 presents comprehensive evaluation metrics for all candidate α values across each economic indicator. Beyond forecast MSE, we utilized a modified Akaike Information Criterion (AIC) adjusted for fractional dynamics

(A1) $$\mathrm{AIC}=-2\ln\left(L\right)+2k+\frac{2k(k+1)}{n-k-1}+C\left(\alpha \right)$$

where *L* represents the likelihood of the data given the model, *k* denotes the number of estimated parameters, *n* is the sample size, and $C\left(\alpha \right)=\lambda {\left(1-\alpha \right)}^{2}$ is a complexity penalty function that increases as α decreases. The AIC results reinforce the optimal range identified through MSE analysis, with the lowest values generally occurring in the α=0.4 to α=0.6 range.

The memory characteristics of each economic indicator were further evaluated through autocorrelation analysis, as illustrated in Figure A7. The autocorrelation functions (ACFs) reveal distinct patterns across indicators: INTEREST shows the slowest decay, indicating the strongest long-range dependence; VIX and CDS exhibit more rapid decay, suggesting weaker memory effects; while CPI and PPI demonstrate intermediate persistence. These patterns are consistent with the estimated Hurst exponents reported in Table A2 and Figure A8, where higher *H* values correspond to slower ACF decay.

**Table A2:** Evaluation metrics for fractional order selection across economic indicators.

Economic Indicator	Metric	α=0.1	α=0.2	α=0.3	α=0.4	α=0.5	α=0.6	α=0.7	α=0.8	α=0.9
INTEREST	Forecast MSE	0.182	0.168	0.158	0.152	0.15	0.152	0.158	0.168	0.182
INTEREST	Hurst Exponent	0.74	0.74	0.74	0.74	0.74	0.74	0.74	0.74	0.74
INTEREST	AIC	352.5	338.0	326.5	318.0	312.5	310.0	310.5	314.0	320.5
CDS	Forecast MSE	0.174	0.163	0.154	0.15	0.15	0.154	0.163	0.174	0.191
CDS	Hurst Exponent	0.76	0.76	0.76	0.76	0.76	0.76	0.76	0.76	0.76
CDS	AIC	348.1	335.2	324.3	316.9	312.5	311.1	313.2	317.3	325.4
VIX	Forecast MSE	0.191	0.174	0.163	0.154	0.15	0.15	0.154	0.163	0.174
VIX	Hurst Exponent	0.65	0.65	0.65	0.65	0.65	0.65	0.65	0.65	0.65
VIX	AIC	357.5	341.3	329.2	319.1	312.5	308.9	308.3	311.2	316.1
CPI	Forecast MSE	0.182	0.168	0.158	0.152	0.15	0.152	0.158	0.168	0.182
CPI	Hurst Exponent	0.82	0.82	0.82	0.82	0.82	0.82	0.82	0.82	0.82
CPI	AIC	352.5	338.0	326.5	318.0	312.5	310.0	310.5	314.0	320.5
PPI	Forecast MSE	0.179	0.166	0.156	0.151	0.15	0.153	0.16	0.17	0.185
PPI	Hurst Exponent	0.79	0.79	0.79	0.79	0.79	0.79	0.79	0.79	0.79
PPI	AIC	350.9	336.9	325.4	317.4	312.5	310.5	311.6	315.1	322.1

The varying rates of autocorrelation decay provide crucial empirical support for our multi-α approach. For instance, VIX with its relatively lower Hurst exponent (H≈0.65) and faster autocorrelation decay is better modeled with α=0.3, which places greater emphasis on long-range memory effects. Conversely, CPI with its higher Hurst exponent (H≈0.82) and slower autocorrelation decay is appropriately captured with α=0.7, which balances recent and historical influences.

While we could have theoretically identified a single “optimal” α value for each economic indicator, such an approach would obscure the rich comparative insights obtained by analyzing information dynamics across different memory assumptions. By employing multiple α values (0.3, 0.5, and 0.7), our framework provides a more comprehensive understanding of how varying memory assumptions affect the information-theoretic properties of these economic indicators. Our α selection methodology combines theoretical foundations with empirical validation to identify fractional orders that effectively capture the spectrum of memory characteristics present in our economic indicators. The results confirm that our selected values of α=0.3, α=0.5, and α=0.7 represent meaningful memory regimes. Specifically, α=0.3 captures strong long-range memory effects particularly suitable for indicators with lower Hurst exponents (e.g., VIX); α=0.5 represents a balanced memory profile with optimal forecast performance across most indicators; and α=0.7 emphasizes more recent dynamics while maintaining some historical dependencies, appropriate for indicators with higher Hurst exponents (e.g., CPI). This multi-α approach allows us to examine how memory assumptions fundamentally alter the information-theoretic characterization of economic interdependencies, offering deeper insights than would be possible with a single fractional order.

## Appendix C. Sensitivity Analysis of γ Parameter

The sensitivity analysis of the numerical regularization parameter γ in the Caputo fractional derivative approximation reveals remarkable stability across multiple orders of magnitude, providing strong empirical validation for our selected value of $\gamma =10^{-6}$. As demonstrated in Figure A9, Figure A10, Figure A11, Figure A12 and Figure A13, we conducted a comprehensive evaluation of γ values ranging from $10^{-8}$ to $10^{-4}$ across fractional orders α∈{0.3,0.5,0.7} for all economic indicators. The heatmaps in the upper panels quantify both mean and maximum percentage differences in CFFI values relative to our baseline choice.

Notably, within the range $10^{-7}\leq \gamma \leq 10^{-5}$, which we designate as the stability interval, the mean percentage differences remain consistently below 0.01%, with most comparisons yielding 0.00% difference to two decimal places. This exceptional numerical consistency is maintained across all fractional orders, demonstrating that our computations are insensitive to moderate variations in the regularization parameter within this range. The maximum percentage difference exhibits similar behavior, with values remaining below 0.03% throughout the stability interval, indicating that even at individual time points where sensitivity might be highest, the impact of varying γ remains negligible.

For values outside this stable range, particularly at $\gamma =10^{-4}$, we observe slightly increased differences (0.01–0.02% in mean difference), though still remarkably small. This confirms our theoretical expectation that larger values of γ begin to artificially dampen the contribution of recent time points in the fractional derivative computation, albeit to a minimal degree within the parameter ranges examined. The consistency of results across fractional orders further suggests that the sensitivity to γ does not substantially increase for smaller values of α, despite the theoretical prediction that the singularity in the kernel becomes more pronounced as α decreases.

The lower panels of the figures provide further visual confirmation of this stability through time-series plots of CFFI values for α=0.5 across three different γ values ($10^{-7}$, $10^{-6}$, and $10^{-5}$). The near-perfect overlap of these trajectories, with the three lines being virtually indistinguishable, demonstrates that the temporal patterns of information flow captured by our CFFI framework remain qualitatively and quantitatively consistent regardless of which γ value is selected within the stability range. The temporal evolution of information spikes, plateaus, and transitions is preserved across all tested values, confirming that the identification of critical information dynamics in our economic indicators is robust to the specific choice of regularization parameter.

## Appendix D. Bandwidth Sensitivity Analysis

The comprehensive bandwidth sensitivity analysis presented in Figure A14, Figure A15, Figure A16, Figure A17 and Figure A18 provides empirical justification for our selection of the bandwidth parameter h=0.25 in the kernel density estimation underlying the CFFI framework. Bandwidth selection represents a fundamental challenge in non-parametric density estimation, requiring careful balance between resolution and stability. Excessively small bandwidths risk amplifying noise when computing fractional derivatives, while overly large values potentially obscure meaningful distributional shifts critical for information-theoretic analysis of economic dynamics.

The mean percentage difference heatmaps reveal striking patterns across all economic indicators, with considerable sensitivity to extreme bandwidth values. Notably, small bandwidths (h=0.10) produce substantially different CFFI estimates compared to our reference value, with deviations exceeding 100% for INTEREST and approaching 200% for CPI. This extreme sensitivity reflects the tendency of small bandwidths to introduce instability in the estimated probability density function, which becomes particularly problematic when computing fractional derivatives. Conversely, larger bandwidths (h≥0.35) also yield significant deviations, though generally of lesser magnitude, typically ranging from 45% to 70% across indicators. The intermediate bandwidth range 0.20≤h≤0.30 demonstrates markedly improved stability, with bandwidth h=0.20 yielding mean percentage differences between 37% and 56% across indicators, substantiating our selection of h=0.25 as a balanced compromise.

Pearson correlation analysis provides further validation of our bandwidth choice, revealing exceptional temporal consistency within the 0.20≤h≤0.30 range. The correlation coefficients in this range consistently exceed 0.98 for all indicators except INTEREST, which nevertheless maintains correlations above 0.98 between h=0.20 and h=0.25. This high degree of correlation confirms that the qualitative patterns of information flow remain remarkably stable across this bandwidth range, despite variations in absolute magnitude. Of particular relevance is the correlation between results at h=0.20 and h=0.25, which exceeds 0.99 for CPI, PPI, and VIX, and remains above 0.98 for CDS. Even at the extremes of our tested range, correlations generally remain substantial (above 0.80 for most indicators), demonstrating the overall robustness of our methodology.

The time-series visualizations of CFFI illustrate how bandwidth affects the amplitude of information spikes while preserving their temporal location. Consistent with theoretical expectations, smaller bandwidths (h=0.20) yield more pronounced peaks, reflecting heightened sensitivity to local distributional changes. This is particularly evident in the VIX series, where amplitudes at critical points (time indices 20, 70, and 100) are approximately 30–40% higher with h=0.20 compared to h=0.25. Conversely, larger bandwidths (h=0.30) reduce peak magnitudes by roughly 25–35% relative to our reference value, indicating greater smoothing of informational transitions. Crucially, the timing of significant information events remains consistent across all bandwidths, confirming that our identification of critical periods is robust to reasonable variations in bandwidth parameter.

The relationship between optimal bandwidth and fractional order α merits particular attention. Examining the correlation patterns across different fractional orders reveals that smaller α values (stronger memory effects) exhibit slightly greater sensitivity to bandwidth choice. For instance, at α=0.3, the correlation between h=0.10 and h=0.25 ranges from 0.641 (INTEREST) to 0.914 (VIX), while at α=0.7, these values improve to 0.607–0.860, reflecting tighter coupling between memory strength and bandwidth sensitivity. This interaction arises because stronger memory effects (smaller α) amplify historical contributions in the fractional derivative, necessitating more careful density estimation to avoid compounding errors.

The optimal bandwidth $h_{opt}$ resulting from our CFFI-specific objective function,

(A2) $$h_{opt}=\arg\min_{h}\left\{\lambda_{1}V_{h}+\lambda_{2}B_{h}\right\},$$

where $V_{h}$ quantifies estimation variance and $B_{h}$ represents bias, yields values consistently centered around 0.25 for most indicators. This aligns with initial estimates derived from established methods such as Silverman’s rule of thumb

(A3) $$h_{ROT}=0.9\min\left(\sigma ,IQR/1.34\right)n^{-1/5}$$

and least-squares cross-validation, which produced values between 0.15 and 0.25. Our refinement through the CFFI-specific objective function, which explicitly accounts for the challenges of differentiating the log-density, provides additional justification for adopting h=0.25 as a universal bandwidth across all indicators.

In conclusion, the bandwidth sensitivity analysis demonstrates that our selection of h=0.25 represents an optimal balance between resolution and stability for CFFI computation across diverse economic indicators. While smaller bandwidths offer enhanced detection of subtle distributional shifts at the cost of increased volatility, and larger bandwidths provide greater smoothing at the expense of attenuated signal strength, the chosen value of h=0.25 preserves critical economic signals while ensuring computational stability. The exceptional correlation values within the 0.20≤h≤0.30 range confirm that our core findings regarding information flow and economic interdependencies remain robust to reasonable variations in this parameter.
